# The NSIGHT1-randomized controlled trial: rapid whole-genome sequencing for accelerated etiologic diagnosis in critically ill infants

**DOI:** 10.1038/s41525-018-0045-8

**Published:** 2018-02-09

**Authors:** Josh E. Petrikin, Julie A. Cakici, Michelle M. Clark, Laurel K. Willig, Nathaly M. Sweeney, Emily G. Farrow, Carol J. Saunders, Isabelle Thiffault, Neil A. Miller, Lee Zellmer, Suzanne M. Herd, Anne M. Holmes, Serge Batalov, Narayanan Veeraraghavan, Laurie D. Smith, David P. Dimmock, J. Steven Leeder, Stephen F. Kingsmore

**Affiliations:** 1 Center for Pediatric Genomic Medicine, Children’s Mercy, Kansas City, MO 64108 USA; 2 Department of Pediatrics, Children’s Mercy, Kansas City, MO 64108 USA; 3 School of Medicine, University of Missouri, Kansas City, MO 64108 USA; 4 Rady Children’s Institute for Genomic Medicine, San Diego, CA 92123 USA; 5 Department of Pediatrics, University of California, Rady Children’s Hospital, San Diego, CA 92123 USA; 6 Department of Pathology, Children’s Mercy, Kansas City, MO 64108 USA; 7 Department of Pediatrics, University of North Carolina, Chapel Hill, NC 27599 USA

## Abstract

Genetic disorders are a leading cause of morbidity and mortality in infants in neonatal and pediatric intensive care units (NICU/PICU). While genomic sequencing is useful for genetic disease diagnosis, results are usually reported too late to guide inpatient management. We performed an investigator-initiated, partially blinded, pragmatic, randomized, controlled trial to test the hypothesis that rapid whole-genome sequencing (rWGS) increased the proportion of NICU/PICU infants receiving a genetic diagnosis within 28 days. The participants were families with infants aged <4 months in a regional NICU and PICU, with illnesses of unknown etiology. The intervention was trio rWGS. Enrollment from October 2014 to June 2016, and follow-up until November 2016. Of all, 26 female infants, 37 male infants, and 2 infants of undetermined sex were randomized to receive rWGS plus standard genetic tests (*n* = 32, cases) or standard genetic tests alone (*n* = 33, controls). The study was terminated early due to loss of equipoise: 73% (24) controls received genomic sequencing as standard tests, and 15% (five) controls underwent compassionate cross-over to receive rWGS. Nevertheless, intention to treat analysis showed the rate of genetic diagnosis within 28 days of enrollment (the primary end-point) to be higher in cases (31%, 10 of 32) than controls (3%, 1 of 33; difference, 28% [95% CI, 10–46%];* p* = 0.003). Among infants enrolled in the first 25 days of life, the rate of neonatal diagnosis was higher in cases (32%, 7 of 22) than controls (0%, 0 of 23; difference, 32% [95% CI, 11–53%];*p* = 0.004). Median age at diagnosis (25 days [range 14–90] in cases vs. 130 days [range 37–451] in controls) and median time to diagnosis (13 days [range 1–84] in cases, vs. 107 days [range 21–429] in controls) were significantly less in cases than controls (*p* = 0.04). In conclusion, rWGS increased the proportion of NICU/PICU infants who received timely diagnoses of genetic diseases.

## Whole genome sequencing: Speedier diagnoses in infants

Genetic disorders in critically ill infants can be diagnosed in as little as 26 h by rapid whole genome sequencing (rWGS). A study led by Stephen F. Kingsmore at the Rady Children’s Institute for Genomic Medicine in San Diego and Children’s Mercy Hospital in Kansas City compared the time to genetic diagnosis in 65 infants with inherited diseases of unknown cause using rWGS, clinical confirmatory testing and standard genetic tests or standard genetic tests alone. They found that the addition of rWGS including confirmatory testing significantly decreased the time to diagnosis, which in newborns can mean the difference between life and death. Because of the increasing accessibility and decreasing costs of the technology and the critical need for timely and effective intervention in infants with suspected genetic diseases, the authors advocate the use of rWGS as a first-line diagnostic test.

## Introduction

A premise of pediatric precision medicine is that outcomes are improved by replacement of clinical diagnosis and empiric management with genetic diagnosis and genotype-differentiated treatment.^1–9^ The evidence base for pediatric precision medicine is still underdeveloped.^10, 11^ Ill infants are especially in need of precision medicine since genetic diseases are a leading cause of mortality, particularly in neonatal intensive care units (NICU) and pediatric intensive care units (PICU).^5–7, 12–16^ Among high-cost health care, NICU treatment is one of the most cost-effective.^17–19^ Since disease progression can be very rapid in infants, genetic diagnoses must be made quickly to permit consideration of precision interventions in time to decrease morbidity and mortality.^5,6, 20–23^ For a few genetic diseases, newborn screening has shown early neonatal diagnosis and rapid, precise intervention to dramatically improve outcomes.^24, 25^ The potential expansion to newborn diagnosis for symptomatic infants for all 5000 genetic diseases^26^ has been made technically possible by the advent of clinical genomic sequencing (whole-genome sequencing (WGS) or whole-exome sequencing (WES), and next-generation sequencing gene panel tests (NGS). In particular, rapid WGS (rWGS) can allow genetic diagnosis in 2 days.^20, 27^

There is substantial evidence that a higher proportion of symptomatic children with likely genetic disease receive etiologic diagnoses by WGS and WES than other genetic tests.^3–7, 28–35^ Published NICU or PICU experience with rWGS, however, is limited to case reports and one retrospective study.^5,6, 20–23^ In the latter, 57% of infants received genetic diagnoses in a median of 23 days (day of life 49).^6^ However, it has not yet been unequivocally demonstrated whether rWGS improves timeliness of genetic diagnosis relative to standard genetic tests. Here we report results of newborn sequencing in genomic medicine and public health randomized controlled trial (RCT) 1 (NSIGHT1), an RCT of genomic testing in patients (ClinicalTrials.gov Identifier: NCT02225522).^24^ Specifically, NSIGHT1 compared rates of genetic diagnosis in NICU and PICU infants with possible genetic diseases at 28 days from enrollment by standard tests alone vs. standard tests plus trio rWGS.

## Results

### Patients

**Fig. 1:**
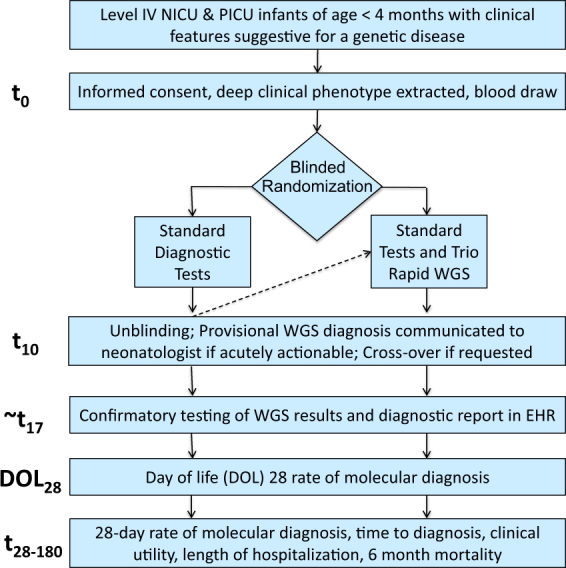
Design of “Newborn Sequencing In Genomic medicine and public HealTh” study 1 (NSIGHT1; ClinicalTrials.gov accession NCT02225522). Time (*t*) is in days. WGS whole-genome sequencing, EHR Electronic Health Record

**Fig. 2:**
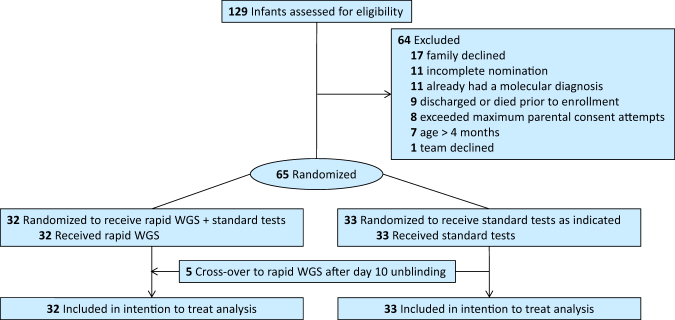
CONSORT flow diagram of NSIGHT1 enrollment and randomization. Major reasons for non-enrollment were family refusal (13%), the infant had a diagnosis that explained the phenotype (9%), and incomplete nominations (9%). At unblinding of clinicians (by 10 days after enrollment), a provision was made whereby clinicians could request compassionate cross-over to the rWGS group if the infant was critically ill. Cross-over was requested for 7 (21%) of 33 infants who randomized to standard tests alone, of which 5 met these criteria and were granted

Of 129 nominated infants, 65 (50%) completed the NSIGTH1 study (Figs. 1 and 2). Sixty four enrollees were NICU infants. The infants nominated represented 7% of NICU and PICU admissions during this interval. Thirty-two infants randomized to rWGS plus standard genetic tests (cases) and 33 to standard tests alone (controls, Figs. 1 and 2). The baseline characteristics of the infants were similar in the two arms and similar to those of a previous retrospective case series of infants receiving rWGS in this NICU and PICU (Table 1).^6^ Detailed (deep) phenotypes of infants were extracted from the electronic medical record in 42 infants receiving genomic sequencing, since this was a prerequisite for interpretation. On average, infants receiving rWGS had 5.9 phenotypic features (range 1–17; Table S1). Phenotypes were highly diverse and typically present at birth (Table 1, S1). The most common indications for nomination were congenital anomalies (35%) and neurological disorders (25%; Table 1). Fewer control infants had cardiovascular findings (6 vs. 28%; difference, −22% [95% CI, −40 to −4%]; *p* = 0.02) than cases, which may have affected likelihood for genetic disease (Table 1).

**Table 1:** Characteristics of the 65 NSIGHT1 probands

		Cases (rWGS, *n* = 32)	Controls (*n* = 33)
Sex	Female (*n*, %)	15 (47%)	11 (33%)
	Male (*n*, %)	16 (50%)	21 (64%)
	Undetermined (*n*, %)	1 (3%)	1 (3%)
Demographics	Caucasian (*n*, %)	25 (78%)	27 (82%)
	African, African American (*n*, %)	2 (6%)	1 (3%)
	Other race (*n*, %)	5 (16%)	5 (15%)
	Hispanic (*n*, %)	2 (6%)	3 (9%)
	Consanguinity (*n*, %)	1 (3%)	2 (6%)
Birth characteristics	Gestational age (average, wks)	36.0	35.9
	Weight (average, kg)	2.5	2.4
	Low birth weight (<2500 g, *n*, %)	14 (44%)	9 (27%)
	Extremely low birth weight (<1000 g, *n*, %)	1 (3%)	3 (9%)
	APGAR at 1 min (average)	6.1	5.1
	APGAR at 5 min (average)	7.8	6.4
	Symptom Onset (average day of life)	2.3	2.1
Primary system involved by disease	Congenital anomalies/musculoskeletal	10 (31%)	13 (39%)
	Neurological	5 (16%)	11 (33%)
	Cardiovascular findings	9 (28%)	2 (6%)
	Endocrine/metabolic	1 (3%)	3 (9%)
	Respiratory findings	4 (13%)	0 (0%)
	Renal	1 (3%)	2 (6%)
	Dermatologic	1 (3%)	0 (0%)
	Multiple system	1 (3%)	0 (0%)
	Hepatic	0 (0%)	0 (0%)
Enrollment and standard clinical tests^a^	Day of life at enrollment (average, range)	22.8 (1–101)	22.0 (1–80)
	Probands receiving standard clinical tests (*n*, %)	30 (93.8%)	33 (100%)
	Day of life 1st standard clinical test ordered (average, range)	11.6 (0–66)	15.6 (0–120)
	All standard clinical tests ordered (average, range)	2.8 (0–7)	3.4 (1–10)
	Probands receiving Standard Clinical NGS panels, WES or WGS (*n*, %)	17 (53%)	24 (73%)
	Standard Clinical NGS panels, WES and WGS Tests Ordered (*n*, range)	22 (0–2)	43 (0–4)
	Probands receiving rWGS^b^	32 (100%)	5 (15%)
Genetic disease diagnoses	Diagnosis (Standard Clinical Test or rWGS; *n*, %)^b^	13 (41%)	8 (24%)
	Diagnosis by Standard Clinical Tests (*n*, %)	7 (22%)	8 (24%)
	Diagnosis by rWGS (*n*, %)^b^	10 (31%)	2 (6%)
	DOL diagnosis by Standard Clinical Test (median, range)	66 (16–151)	130 (37–451)
	Time to Diagnosis by Standard Clinical Test (average, range)	45 (16–150)	110 (31–450)

^a^The statistics for standard clinical tests exclude newborn screening, which all infants received, and did not result in any diagnoses

^b^Includes Controls 5007, 5012, 5029, 5040 and 5053, which were crossed over to rWGS

### Standard diagnostic tests

Standard diagnostic tests for genetic diseases were performed as clinically indicated in 63 of the 65 infants (Table 1). They included all postnatal diagnostic tests that could be ordered through the electronic medical record. The proportion of infants receiving standard genetic tests and age at first standard test order were similar in both arms (Table 1). In addition to newborn screening infants received an average of 3.1 (range 0–10) standard genetic tests (Table 1, S3), which was similar to a previous retrospective case series of infants receiving rWGS from the same NICU and PICU.^6^ During the study, non-expedited WGS became available as a standard diagnostic test. Of 33 control infants, 24 (73%) received non-expedited clinical NGS panel tests, WES or WGS standard tests, compared with seventeen (53%) of 32 cases (Table 1, S3). Other than newborn screening, the average age at first standard test order was 14 days (range 0–120 days). Standard tests yielded fifteen (24%) genetic diagnoses in the 63 subjects tested, seven (23%) in 30 cases, and eight (24%) in 33 controls (Table 2, S4). The rates of diagnosis by individual standard clinical tests were: chromosomal microarray 6% (three of 48 tests); Clinical NGS panel test 18% (nine of 49 tests); Clinical WES 33% (one of three tests); Methylation 13% (one of eight). Of note, five (33%) of 15 diagnoses by standard tests would not have been detected by rWGS at the time of study: four were copy number or structural variants and one was a change in DNA methylation. The median time from first standard test order to diagnosis was 64 days (range 16–450 days). The average age at diagnosis by standard genetic tests was 113 days (range 16–451 days). Six (10%) of 63 infants received a diagnosis by standard tests prior to hospital discharge (Table S5).

**Table 2:** Presentations and characteristics of the twenty one infants who received diagnoses (Dx) of genetic diseases

Patient ID	Study arm	Dx type	Mode of Dx	Primary clinical features^a^	Diagnosis	Gene	Inheritance pattern	De novo or inherited	Variant chromosomal (Chr)^b^ or gene (c.) coordinate	Variant patho-genicity	Variant protein coordinate
5004	Case	Partial	Std	Cleft palate micrognanthia hypoglycemia hyperinsulinimia thrombocytopenia	Chr 7p duplication syndrome	n.a.	n.d.	n.d.	Gain 7p22.3-p15.2 Chr7:43360-26463160dup	P	n.a.
5007	Control	Full	rWGS and Std	Polymicrogyria intractable seizures epileptic encephalopathy	Congenital disorder of glycosylation type Ik	*ALG1*	Autosomal Recessive	Inherited	c.15 C > A and c.149 A > G	P and LP	p.C5* and p.Q50R
5008	Case	Full	Std	Complete atrioventricular canal defect hypospadias IUGR dysmorphic features	Chr 8p23 deletion syndrome	n.a.	n.d.	n.d.	Chr8:158048-6999114del 10054927-10479436dup 10479473-11882401del	P	n.a.
5011	Control	Full	Std	Hypotonia cryptorchidism aniridia	XL myotubular myopathy-1	*MTM1*	X-Linked Recessive;	n.d.	c.137-3 T > G;	P	n.a.
					Aniridia	*PAX6*	Autosomal Dominant	Inherited	c.1268 A > T	P	p.*423 L
5014	Control	Full	Std	Hyperglycemia	Transient neonatal diabetes	*ZFP57*	n.d.	n.a.	Hypomethylation 6q24	P	n.a.
5023	Case	Full	rWGS and Std	Hyponatremia SGA/IUGR pseudohypoaldosteronism	Pseudohypoaldosteronism type I	*NR3C2*	Autosomal Dominant	Inherited	c.1951C > T	LP	p.R651*
5025	Control	Full	Std	Micrognathia cleft palate abnormal facies right thumb hypoplasia	Nager type acrofacial dysostosis	*SF3B4*	Autosomal Dominant	de novo	c.1088-3 C > G	LP	n.a.
5026	Control	Full	Std	Hirsutismmild synophrys mild micrognathia camptodactyly renal cysts	Cornelia de lange syndrome 1	*NIPBL*	Autosomal Dominant	de novo	c.5057del	P	p.L1686Rfs*7
5027	Control	Full	Std	IUGR cleft palate Micrognathia Skin tags Poor gag reflex	Chr 1p36 deletion syndrome	n.a.	Autosomal dominant	de novo	Loss arr 1p36.11 Chr1:24100645-25003678del	LP	n.a.
5030	Case	Full	Std	Seizures poor feeding	AD nocturnal frontal lobe epilepsy	*CHRNA4*	Autosomal Dominant	de novo	Heterozygous deletion of CHRNA4	LP	n.a.
5035	Case	Full	rWGS	Microcephaly	Primary AR microcephaly 5	*ASPM*	Autosomal Recessive	Inherited	c.3428dupT; c.8191_8192del	P,P	p.L1144Vfs*16; p.E2731Kfs*19
5036	Case	Full	rWGS	Central apnea	Congenital central hypoventilation syndrome	*PHOX2B*	Autosomal Dominant	de novo	PHOX2B ALA EXP	P	p.A260(9)
5038	Case	Full	rWGS	Situs inversus	Primary ciliary dyskinesia type 7	*DNAH11*	Autosomal Recessive	Inherited	c.6244 C > T; c.6776 A > T and c.8567 T > C	P, LP, LP	p.R2082*; p.D2259V and p.V2856A
5042	Case	Full	rWGS	Profound hypotonia Respiratory distress Myoclonic jerks	AD mental retardation 31	*PURA*	Autosomal Dominant	de novo	c.458_459dupC	P	p.K154Qfs*47
5048	Case	Full	rWGS	Seizures	Early infantile epileptic encephalopathy 14	*KCNT1*	Autosomal Dominant	de novo	c.1420 C > T	P	p.R474C
5051	Case	Full	rWGS	Perinatal ascites; cholestasis	Dehydrated hereditary stomatocytosis	*PIEZO1*	Autosomal Dominant	Inherited	c.6058 G > A	P	p.A2020T
5053	Control	Full	rWGS and Std	Altered mental status Decreased deep reflexes Hypotonia cryptorchidism	XL myotubular myopathy	*MTM1*	X-linked Recessive	de novo	c.567_569delTAA	P	p.N189del
5057	Case	Full	rWGS and Std	Dysmorphic features Cardiac anomalies failed hearing screen	Noonan syndrome	*SOS1*	Autosomal Dominant	de novo	c.2536 G > A	P	p.E846K
5059	Case	Full	rWGS and Std	HLHS hydrocephalus multiple congenital anomalies	Coffin–siris syndrome	*ARID1A*	Autosomal Dominant	de novo	c.1207 C > T	LP	p.Q403*
5061	Case	Partial	rWGS and Std	Hypotonia absent gag reflex exaggerated startle reflex	Hyperekplexia	*GLRA1*	Autosomal Dominant	de novo	c.373 G > A	LP	p.D125N
5062	Control	Full	Std	Bicuspid aortic valve, hypotonia, leukocytosis	Central core disease of muscle	*RYR1*	Autosomal Dominant	de novo	c.14581 C > T	P	p.R4861C

Controls 5007, 5012, 5029, 5040 and 5053 were crossed over to rWGS

*Chr* chromosome, *std* standard genetic test, *P* pathogenic, *LP*likely pathogenic, *n.d.* not determined

*Premature stop codon created

^a^Full clinical features are shown in Table S1

^b^GRCh37

### Rapid whole-genome sequencing

rWGS was performed on infant-parent trios with Illumina HiSeq instruments, with paired reads to an average depth of 40-fold, detecting an average of 5.0 million nucleotide variants per genome (standard deviation 0.3 million variants; Table S2, Figure S1).

Ten of 32 cases (31%) received diagnoses by rWGS (Table 2, Table S4). Upon un-blinding of clinicians to randomization at day 10 after enrollment, compassionate cross-over to rWGS was requested for seven (21%) of the 33 controls. Cross-over to rWGS was declined in two infants who were not acutely ill; both were about to be discharged to home, with follow-up of their medical conditions as outpatients. Five cross-over requests were granted, yielding two diagnoses. In both, diagnosis by rWGS occurred first but was recapitulated by standard tests (Table 2). Including five crossovers, 12 (32%) of 37 infants received rWGS diagnoses (Table 2, S5). On average, enrollment occurred on DOL 22 (range 1–101; Table 1), which was earlier than in our previous report of rWGS (DOL 26; Table 1),^6^ but an average of 8 days later than standard tests. The median time to rWGS diagnosis, including clinical confirmatory testing, was 14 days (range 8–35 days; Table S5), which was also faster than our previous report of rWGS (23 days; Table S5).^6^ The median age at diagnosis in patients randomized to rWGS was 28.5 days (range 14–90 days). Among crossovers, the median age at rWGS diagnosis was 94.5 days.

The research protocol required confirmation of rWGS results by another method prior to clinical reporting except in cases where life-threatening progression was imminently likely. There were no such cases, and no provisional diagnostic reports of rWGS results were returned prior to confirmatory testing. Sanger sequencing confirmed all rWGS results.

### Diagnoses

Twenty-two genetic diagnoses were reported in 21 (32%) of 65 infants (Table 2). Thirteen cases (41%) received diagnoses by rWGS or standard tests. Eight controls (24%) received diagnoses (Table 1). One individual received two diagnoses. Only one diagnosis was recurrent (X-linked myotubular myopathy in two infants), reflecting substantial genetic heterogeneity among NICU disease presentations^6^ (Table 2). The most common mechanism was de novo variant occurrence (12 of 21 (57%) diagnoses; Table 2). Seventeen (65%) causative variants were reported as pathogenic, and nine (35%) as likely pathogenic. Eight variants (31%) were predicted to result in amino acid substitutions, six (23%) were indels, five (19%) were predicted to result in stop codon loss or gain, four (15%) were structural variations, two (8%) were predicted to alter splicing, and one (4%) impacted methylation. The most common inheritance pattern was autosomal dominant (14 of 19 (74%) diagnoses), followed by autosomal recessive (three, 16%) and X-linked recessive (two, 11%). In 19 of 21 (90%) diagnosed probands, the diagnosis explained all of the clinical features (Table 2). Two possible diagnoses provoked discussion regarding inclusion. Control infant 5053 had altered mental status, decreased deep tendon reflexes, hypotonia, and cryptorchidism (Table S1). He had a duplication of Chr 1p36.32 as well as X-linked Myotubular Myopathy. Chr 1p36.32 duplication syndrome was not included as a diagnosis. The second, paternally inherited *SCN5A* c.6010 T > C (p.F2004L) in Case infant 5033 with persistent, symptomatic atrial fibrillation/flutter (Table S1), was not included as a diagnosis. While several case studies had reported this variant as pathogenic and two functional studies showed it to be deleterious, the allele frequency (0.3%) was considered too high to be likely pathogenic, and a diagnosis of familial atrial fibrillation type 10 was not reported.^36–40^

### Clinical utility of molecular diagnoses

The short-term clinical impact of diagnoses was assessed by chart reviews and surveys with referring physicians (Table S4). Thus, clinical utility reflected actual practice, rather than an ideal or maximal personalization of treatment. Clinical utility did not include the impact of negative test results on management. 20 (31%) of the 65 infants enrolled (95% of those receiving diagnoses) had a consequent change in management. Ten (15%) infants (48% of those receiving diagnoses) had a change in management other than genetic or reproductive counseling of parents. Diagnoses were not associated with any harms.

### Early study termination

The study was terminated after 21 months due to growing availability of targeted NGS panels, WES and WGS as standard tests, which shifted the baseline of comparison over the course of the study. These were associated with high rates of cross-over requests and higher utilization of targeted NGS panels, WES or WGS as standard clinical genetic tests among controls (73%) than cases (53%; Table S3).

### End-point testing

**Fig. 3:**
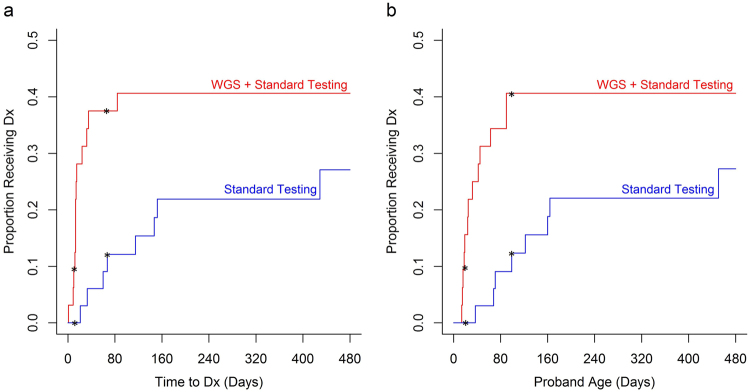
Kaplan–Meier curves of time to diagnosis in cases and controls. The cumulative probability of a diagnosis (Dx) in cases (infants randomized to receive rWGS plus standard genetic tests; shown in red; *n* = 32) and controls (infants randomized to standard genetic tests alone; shown in blue; *n* = 33). Differences in probability of receiving a diagnosis were significant between the two arms from day 12–67 after enrollment (**a** asterisks) and DOL 19 - 99 (**b** asterisks)

End-points were analyzed on the basis of intention to treat (Figs. 1 and 2). Thus all patients were analyzed in the groups to which they were randomized. The primary end point, rate of genetic diagnosis within 28 days of enrollment, was higher in cases (31%, ten of 32) than controls (3%, one of 33; difference, 28% [95% CI, 10–46%]; *p* = 0.003 Table 3). Kaplan–Meier curves supported the conclusion that there was a significantly higher probability of receiving a diagnosis by rWGS until DOL 99 or 67 days after test order (Fig. 3). For neonates enrolled within the first 25 days of life, the rate of diagnosis by DOL 28, a secondary end-point, was higher in cases (32%, seven of 22) than controls (0%, zero of 23; difference, 32% [95% CI, 11–53%]; *p* < 0.01; Table 3). In practice, crossovers did not materially affect these end-points, since the two diagnoses made by rWGS among five cross-over infants occurred later than DOL 28 and 28 days after enrollment (Table S3).

**Table 3:** Comparison of primary and secondary end-points

	rWGS + standard testing	Standard testing (including crossovers)	*P*-value	Statistical test
Number of subjects	32	33		
Primary end-point				
Diagnosis within 28 days of enrollment (*n*, %)	10 (31%)	1 (3%)	0.003^a^	Fisher’s exact test
Secondary end-points				
Diagnosis by DOL 28 (*n*, %)	7 (32%)	0 (0%)	0.004^a^	Fisher’s exact test
Total diagnoses (*n*, %)	13 (41%)	8 (24%)	0.19	Fisher’s exact test
Clinical utility of diagnoses (*n*, %)	13 (41%)	7 (21%)	0.11	Fisher’s exact test
DOL hospital discharge (average, range)	66.3 (3–456)	68.5 (4–341)	0.91	Two sample *t*-test
Diagnosis before discharge (*n*, %)	9 (28%)	3 (9%)	0.06	Fisher’s exact test
Mortality at 180 days (*n*, %)	4 (13%)	4 (12%)	n.d.	
Age at death (days; median, range)	62 (14–228)	173 (4–341)	0.93	Log rank test

^a^Fisher’s exact test *p*-value both for all patients and in a sensitivity analysis, in which patients with a partial diagnosis (5004 and 5061) where considered undiagnosed

*DOL* day of life

Age at diagnosis and time to diagnosis differed significantly between arms, after accounting for non-proportional rates of diagnosis (Table 4, Table S5): The median age at diagnosis in cases was 25 days (range 14–90 days) vs. median in controls was 130 days (range 37–451). The median time to diagnosis in cases was 13 days (range 1–84 days) vs. median in controls 107 days (range 21–429 days). Two diagnoses explained part of the infant’s disorder rather than the entire presentation. In a sensitivity analysis, when patients adjudged to have a partial diagnosis were considered undiagnosed, age at diagnosis and time to diagnosis were no longer significant (Table 4).

**Table 4:** Comparison of age at diagnosis and time to diagnosis between cases (rWGS plus standard tests) and controls (standard tests alone)

	Original analysis^a^		Sensitivity analysis^b^	
	*p*-value for non-proportional hazards	*p*-value for a difference in overall diagnostic rates	*p*-value for non-proportional hazards	*p*-value for a difference in overall diagnostic rates
Age at diagnosis	0.002	0.043	0.003	0.15
Time to diagnosis from enrollment	0.002	0.040	0.002	0.11

^a^Peto–Peto test used instead of log-rank test due to evidence of non-proportional hazards

^b^Peto–Peto test when patients with a partial diagnosis (5004 and 5061) considered undiagnosed.

Six other secondary end-points did not differ significantly between arms in an intention to treat analysis (Tables 1, 3, 4, S4). They were the proportion of infants receiving diagnoses of genetic diseases (41% of cases vs. 24% of controls; difference, 16% [95% CI, −6 to 39%])), proportion in whom diagnoses had clinical utility (41% of cases vs. 21% of controls; difference, 19% [95% CI, −3 to 42%]), proportion of infants with a change in medical management (clinical utility, 22% of cases vs. 9% of controls; difference, 13% [95% CI, −5 to 30%]), proportion of patients who received diagnoses prior to hospital discharge (28% of cases vs. 9% of controls; difference, 19% [95% CI, 0–38%]), average length of NICU/PICU stay (average 67 days), 6-month mortality (12%, 8 of 65), and age at death.

## Discussion

NICU and PICU infants receiving trio rWGS plus standard clinical testing had a higher rate of genetic diagnosis and shorter time to diagnosis than infants receiving standard tests alone. In intention to treat analysis, rWGS was associated with significantly more genetic diagnoses within 28 days of enrollment (31%, 10 of 32) than standard tests alone (3%, 1 of 33; difference, 28% [95% CI, 10–46%]; *p* = 0.003). The rate of neonatal (DOL 28) diagnosis was higher in cases (32%, 7 of 22) than controls (0%, 0 of 23; difference, 32% [95% CI, 11–53%]; *p* = 0.004). Of note, standard genetic testing was ordered an average of 8 days before enrollment, which benefitted the control arm over rWGS cases for these analyses. Nevertheless, age at diagnosis and time to diagnosis were significantly shorter in rWGS cases, after accounting for non-proportional rates of diagnosis.

The rate of genetic diagnosis by rWGS in a NICU or PICU was reported previously in one cohort.^6^ Enrollment in that study was at average DOL 26 (vs. DOL 22 herein). The rate of diagnosis by rWGS therein was 14% (5 of 35) by DOL 28, and 34% (12 of 35) within 28 days of enrollment, which were similar to herein (32% and 31%, respectively). The total rate of genetic diagnosis by rWGS herein (32%) was within the range reported for WGS and WES studies.^3–7, 28–35^

Timely return of rWGS diagnoses was limited by two research factors that may not be part of routine clinical practice: firstly, confirmatory testing by “the clinically accepted standard” was required for research rWGS diagnoses—but was not necessarily required for laboratory developed, clinical WGS, WES, and targeted NGS panel tests—which lengthened the time to rWGS diagnosis by 7–10 days. Indeed, all diagnostic rWGS findings in the current study were concordant with orthologous methods. For well covered, pathogenic and likely pathogenic, single nucleotide variants in regions of high WGS quality, a median time-to-result of 5 days is anticipated.^6,20, 27^ Secondly, enrollment occurred relatively late during the NICU or PICU stay (DOL 22). While parents are interested in receipt of genomic sequencing at birth, an enrollment rate of 6% was reported for WES in NICU infants in another cohort.^41, 42^ Delay in enrollment herein reflected two logistical factors. First, since a criterion for enrollment was suspicion by the provider of an underlying genetic disease, nomination was often delayed until a genetic test or consult had been ordered. In such cases, the time of enrollment delayed the study test, rWGS, compared to standard testing; nevertheless, there was still a decreased time to diagnosis with rWGS. Secondly, NSIGHT1 required informed consent from both parents; the logistics and complexity of obtaining informed consent in a NICU or PICU setting are arduous. A follow-on study, NSIGHT2, has started in which enrollment occurs close to the day of NICU or PICU admission (ClinicalTrials.gov Identifier: NCT03211039). This was facilitated by simpler enrollment criteria, requirement of informed consent from a single parent, and limiting eligibility for enrollment to within several days of admission.

Since the current study, clinical rWGS has improved with respect to rate of genetic diagnosis and time to diagnosis.^27^ In particular, the diagnostic rate has increased through ongoing identification of novel disease genes, improved reference genome sequences, and better identification of disease-causing copy number, repeat expansion, regulatory, splicing and structural variations.^32, 43–50^ These recent advances were not reflected in the current study. Indeed, in three cases herein, causative chromosomal deletions were detected by microarray but not by rWGS. rWGS has recently also become much more feasible in clinical laboratories due to improved throughput of rapid sequencing instruments (Illumina NovaSeq 6000), and the availability of robust commercial interpretation software. Rapid trio exome sequencing (rWES) has also become feasible, and has demonstrated similar performance to rWGS^51^: In a recent study, rWES revealed a molecular diagnosis in 51% of infants at an average of 33 days of life, and with a mean turnaround time of 13.0 days.^51^ Randomized, controlled studies are needed that compare the diagnostic and clinical utility and cost effectiveness of rWES and rWGS in NICU and PICU infants

NSIGHT1 was terminated early, primarily due to loss of equipoise noted by some nominating clinicians during the study. Some practitioners grew to regard randomization to standard tests alone to be an inferior intervention than standard tests plus trio rWGS. This was associated with seven (21% of controls) requests to cross-over control infants to the rWGS arm following clinician un-blinding, five of which were granted. It was also associated with a higher rate of order of targeted NGS panels, WES or WGS standard genetic tests in controls (43 tests in 24 controls) than cases (22 tests in 17 cases). Standard genomic sequencing tests accounted for 63% (5) of the eight genetic diagnoses in controls. As a result, there was not a significant difference between arms in the total number of genetic diagnoses, a secondary end-point (41% [13] diagnoses among 32 infants in the rWGS arm, 24% (8) of 33 in controls; difference, 16% [95% CI, −6 to 39%]; *p* = 0.19). Future pragmatic RCT designs in genomic medicine will require careful attention to the principle of equipoise and to the rapid evolution of clinical NGS-based testing.^52, 53^ The more widespread use of gene panel testing in the NICU during the course of this study was a significant departure from our experience at study conception. Our study was not intended to evaluate the relative diagnostic yield of panel testing over rWGS. Consequently, the study was not powered to evaluate the non-inferiority of panels over rWGS.

The rationale for rWGS in NICU infants is to enable consideration of acute precision interventions in time to decrease morbidity and mortality.^5,6, 21–24^ In two prior studies of genomic sequencing in infants, genetic diagnoses led to precision medicine that was considered life-saving in 5%, and that avoided major morbidity in 6%.^6, 7^ In those studies, early diagnosis (DOL 49) led to greater implementation of precision medicine (65%) than later diagnosis (DOL 374, 39%), particularly with regard to palliative care guidance. As in the current study, assessments of clinical utility were based on actual changes in management, which were limited by clinician experience with genomic medicine and rare genetic diseases. This is a major challenge for NICU and PICU implementation of genomic medicine for rare genetic diseases.^54, 55^ Unfortunately, early termination of the current study resulted in loss in power for the secondary end-points: there were not significant differences in the overall rate of clinical utility of diagnoses, length of admission, rate of diagnosis before discharge, mortality and age at death. The clinical utility of diagnoses and rate of diagnosis before hospital discharge trended towards being higher in the rWGS arm (difference, 19% [95% CI, −3 to 42%], *p* = 0.11, and 19% [95% CI, 0–38%], *p* = 0.06, respectively). Additional studies are needed to clarify whether shorter time to diagnosis is associated with changes in clinical utility of diagnoses, outcomes, or healthcare utilization.

## Conclusions

Among infants with suspected genetic diseases in a regional NICU or PICU, the addition of rWGS decreased the time to diagnosis. Since genetic diseases are among the leading cause of death in the NICU and PICU, as well as overall infant mortality, implementation of rWGS is likely to have broad implications for the practice of neonatology.

## Methods

### Trial design

NSIGHT1 tested the a priori hypothesis that rWGS increases the proportion of infants receiving a genetic diagnosis within 28 days in a partially blinded, randomized controlled study in a regional NICU and PICU in a tertiary referral children’s hospital (Children’s Mercy—Kansas City, CM-KC)(Fig. 1). Infants were born at CM-KC or transferred from outside birthing hospitals to CM-KC for intensive care at various ages. Inclusion criteria were infants in the NICU or PICU of age less than four months with illnesses of unknown etiology and one of the following: 1. A genetic test order or genetic consult; 2. A major structural congenital anomaly or at least three minor anomalies; 3. An abnormal laboratory test suggested a genetic disease; or 4. An abnormal response to standard therapy for a major underlying condition. Exclusion criteria were a previously confirmed genetic diagnosis that explained the clinical condition, or features pathognomonic for a chromosomal aberration. The NICU census was reviewed daily for eligible infants by enrollment coordinators. The eligibility criteria did not change after trial commencement. NICU clinicians were notified of eligible infants, who were nominated through a standard form. NICU and PICU clinicians notified families of eligible infants about the study, and enrollment coordinators then approached parents for informed consent. Enrolled infants were randomly assigned in a 1:1 ratio to receive standard, clinically determined tests (controls) or standard clinical tests plus trio (infants and parents where available) rWGS for etiologic diagnosis of infants’ underlying conditions (cases; Fig. 1). Randomization was performed automatically by the RAND function in Microsoft Excel at enrollment. Parents and clinicians were initially blinded. However, by day ten they were notified of randomization assignment, to minimize parental anxiety and allow consideration of crossover to rWGS. The study design was adaptive, with modification of enrollment prospectively planned following interim data analysis after approximately 2 years of accrual.

### Sample size determination

The study proposed a sample size of 500 in each group (1000 total), with 82% power to detect a difference of 0.05 in the proportion of molecular diagnoses (using a case diagnosis proportion of 0.1 and a control diagnosis proportion of 0.05; two group, continuity-corrected *χ*^2^ test with a 0.05 two-sided significance level). We assumed that both primary outcome groups were independent, and contained 500 subjects. We are also interested in comparing the mean time to molecular diagnosis among the two independent study arms. Assuming that 5% of control subjects and 10% of those receiving rWGS receive a molecular diagnosis, the study had more than 98% power to detect the difference in mean time to molecular diagnosis between the cases and controls (96 ± 24 h in rapid whole-genome sequencing cases vs. 240 ± 72 h in controls; two group Satterthwaite *t*-test with a 0.05 two-sided significance).

### Ascertainment of clinical features and study measures

The clinical features of affected infants receiving rWGS were ascertained comprehensively by review of the electronic medical record and discussion with physicians and entered in a study REDCap database.^56^ Phenotypic features were translated into Human Phenotype Ontology terms and mapped to ~5000 monogenic diseases with the clinicopathologic correlation tools SSAGA, Phenomizer and Phenolyzer, generating rank-ordered, deep differential diagnosis lists^27,57, 58^ (Table S1). Baseline demographics including age, gender, gestational age, birth weight, APGAR scores, and family history were collected. Other study measures were entered into the REDCap database, including diagnostic tests ordered during hospitalization, changes in clinical management following diagnostic test reporting, length of hospitalization, and mortality. Enrollment was from October 2014 to June 2016, and data collection continued until November 2016.

### Trial oversight

The investigators designed the trial in consultation with NICU and PICU staff and program managers of the funding agencies, the National Human Genome Research Institute (NHGRI) and Eunice Kennedy Shriver National Institute of Child Health and Human Development (NICHD).^24^ The investigators received a pre-submission opinion from the Food and Drug Administration (FDA), Center for Devices and Radiological Health (CDRH), Office of In Vitro Diagnostics and Radiological Health (OIR), that the study posed a nonsignificant risk for enrollees, and did not need to be performed under an Investigational Device Exemption (FDA/CDRH/OIR submission Q140271, May 8, 2014). The study was approved by the Institutional Review Boards at Children’s Mercy—Kansas City (CM-KC) and Rady Children’s Hospital, San Diego, and conducted in accordance with the Declaration of Helsinki. Data were collected and analyzed by the investigators. All authors participated in the writing of the manuscript and approved the draft that was submitted for publication. The funding sources were not involved in the collection, analysis, or interpretation of the data, or the writing of the report. The first draft of the manuscript was written by the corresponding author. The authors vouch for the accuracy and completeness of the data and data analyses and for the fidelity of the trial to the protocol (ClinicalTrials.gov accession NCT02225522).

### Rapid whole-genome sequencing

rWGS was performed under a research protocol and employed 26-h–7-day methods, guided by acuity of illness of the proband as described.^5,6,20, 27^ When possible, rWGS was performed on specimens from both biological parents and affected infants simultaneously. Of 37 infants receiving rWGS, 31 were analyzed as trios, 3 as mother-infant duos, 2 as singletons, and 1 as a quad with two affected siblings. Genomic DNA was prepared for rWGS using either TruSeq PCR Free (Illumina, San Diego) or KAPA HYPER (KAPA Biosystems), and resultant libraries were quantified by real-time PCR. WGS was with the Illumina HiSeq 2500 (v4 chemistry, 2 × 125 or 2 × 101 nucleotides, nt) in rapid run or high output mode, or HiSeq 4000 (2 × 125 nt). rWGS was to a minimum depth of 90 Gb per genome, and the average genome coverage was 40-fold (Table S2, Figure S1). All samples met established quality metrics.

### Rapid WGS analysis and diagnostic interpretation

rWGS were generated with Illumina RTA 1.12.4.2 and CASAVA-1.8.2, and aligned to the human reference genome GRCh37.p5 using GSNAP and bwa-mem v0.7.12. Nucleotide (nt) variants were detected and genotyped with the Genome Analysis Tool Kit (v1.6-13. and v3.2-2).^5,6,20, 27^ Copy number variants and structural variants were not detected in WGS. Nucleotide variants were annotated with the Rapid Understanding of Nucleotide variant Effect Software (RUNES).^5,6, 20–27^ Variants were interpreted by board certified molecular geneticists using American College of Medical Genetics guidelines for pathogenic and likely pathogenic classifications.^59^ Causative variants were identified primarily with Variant Integration and Knowledge INterpretation in Genomes (VIKING) software.^5, 6^ Inputs for VIKING were the annotated genomic variant file produced by RUNES and a Phenomizer file of the genes on the comprehensive differential diagnosis.^5, 6^ Alternatively, diagnostic searches utilized pre-calculated candidate gene lists, such as genes with OMIM records or genes associated with mitochondrial disorders. Interpretation considered multiple sources of evidence, including variant pathogenicity, inheritance pattern, strength of disease–gene association, and match of the clinical features of the disease with a deep patient phenotype. All inheritance patterns were examined. Analysis was performed sequentially by two experts. Where a single likely causative variant for a recessive disorder was identified, the locus was manually inspected using the Integrated Genome Viewer in the trio for uncalled variants.^60^ Expert interpretation and literature curation were performed for likely causative variants with regard to evidence for pathogenicity. The FDA and IRB approved return of verbal, provisional rWGS diagnosis to the treating physician in exceptional cases, where the results were actionable and the infant was imminently likely to die or have worsening morbidity. Familial relationships were confirmed by segregation analysis of variants. All diagnostic genotypes were confirmed by Sanger sequencing prior to final reporting. rWGS and Sanger sequencing were performed in a laboratory licensed by the Clinical Laboratory Improvement Amendments and accredited by the College of American Pathologists. Additional expert consultation and functional confirmation were performed in selected cases when the subject’s phenotype differed from previous mutation reports for that disease gene or when the pathogenicity of variants was uncertain. In the absence of a diagnosis, a research note was placed in the medical record to indicate that testing was complete. At time of study performance, clinical grade detection of copy number and structural variants was not performed. Secondary and incidental findings were not reported.

### Standard genetic testing

Standard clinical testing for genetic disease etiologies was performed in infants based on physician clinical judgment, assisted by subspecialist recommendations. Specimens for all standard tests were collected and transported as quickly as possible, and all standard tests were performed as expeditiously as possible. The set of genetic tests considered to be standard was developed by three pediatrician subspecialists. Standard genetic tests were those ordered through the electronic medical record, and included biochemical and immunologic testing for genetic diseases, array comparative genomic hybridization, fluorescence in situ hybridization, high resolution chromosomes analysis, Sanger sequencing of genes, non-expedited proband targeted next-generation sequencing (NGS) gene panels, non-expedited proband whole-exome sequencing (WES), non-expedited proband WGS, methylation studies, and gene deletion/duplication assays (Table S3), as well as Kansas or Missouri state newborn screening (including five lysosomal storage diseases).

### Trial end points

The pre-specified primary end point was a comparison of the proportion of patients receiving a molecular diagnosis within 28 days of enrollment. The pre-specified secondary end points were the proportion of patients receiving a molecular diagnosis within the neonatal period (28 days of life), total diagnostic rate, time-to-diagnosis, and percentage of patients with a change in management related to test results in the two arms. Other pre-specified end-points were the length of hospitalization and short term mortality rate. Data related to these end-points were entered into the REDCap database and audited by two study investigators. The end-points were not changed after the trial commenced. Change in management was determined by a survey of nominating clinicians and review of the electronic health record by at least two pediatrician subspecialists with substantial expertise in genomic medicine to identify change in treatments and procedures, canceled tests, new focused tests, recommendation for specific follow-up related to the diagnosis, and changes in consultation related to the diagnosis.^61^ A modified Delphi method was used to determine inclusion of change in management where there was disagreement.

### Statistical analysis

Statistical analyses were based on the intention-to-treat principle to avoid confounding due to the crossover of controls to the rWGS group. Variables representing whether patients received a diagnosis and whether they received a change in care were treated as dichotomous (y/n). Controls were considered diagnosed only if they received a molecular diagnosis from standard tests regardless of whether they were crossed over and received a diagnosis from rapid WGS. Cases were classified as diagnosed if they received at least one diagnosis from either rapid WGS or standard tests.

Primary analyses comparing 28-day diagnostic rates between study arms were conducted using Fisher’s exact test. Differences in secondary endpoints, including total diagnoses, clinical utility of diagnoses, and diagnoses before discharge, were also assessed with Fisher’s exact test. A two-sample *t*-test was performed to evaluate the null hypothesis of no difference in mean age at hospital discharge for rapid WGS cases and controls.

Kaplan–Meier analyses were used to compare time to diagnosis for each study arm. We generated plots of the cumulative diagnostic rates, estimated as one minus the Kaplan–Meier function, and 95% confidence intervals. For the analysis of age at diagnosis, time was measured from date of birth to date of diagnosis. For the analysis of time to diagnosis from first test, time to diagnosis was measured from the date of enrollment. Patients that did not receive a diagnosis by the end of the study had their data censored at the final date of data collection (November 2016). In general, the log-rank test is most powerful in the presence of proportional hazards. Thus for age at death, for which there was no evidence of non-proportional hazards, the log-rank test was performed.^62^ When there was evidence of a non-constant hazard ratio, between-group differences were evaluated with the Peto-Peto test.^63, 64^ The latter was used as an alternative to the log-rank test when comparing time to diagnosis between WGS plus standard tests and standard tests alone because of its increased power, especially when differences are hypothesized to occur early in time.

As a sensitivity analysis, we reclassified patients who received a partial diagnosis as undiagnosed and repeated analyses for relevant primary and secondary end-points. All reported *p*-values are two-sided and were considered statistically significant if less than 0.05. Statistical analyses were performed in R version 3.3.0.^65^

### Data and material availability

Data are available at LPDR (https://www.nbstrn.org/research-tools/longitudinal-pediatric-data-resource).

## Key points

Question: What proportion of acutely ill inpatient infants receive a diagnosis of a genetic disease within 28 days with rapid whole-genome sequencing?

Findings: In a RCT of 65 infants, the diagnostic sensitivity of rapid whole-genome sequencing within 28 days was 31 vs. 3% with standard genetic testing, a significant difference.

Meaning: In NICU and PICU infants with diseases of unknown etiology, rapid whole-genome sequencing may be warranted as a first-line diagnostic test.

## Electronic supplementary material

Supplementary Material 1
