# Supplementary material for: The NSIGHT1-randomized controlled trial: rapid whole-genome sequencing for accelerated etiologic diagnosis in critically ill infants
Source: NPJ Genom Med. 2018 Feb 9;3:6. doi: 10.1038/s41525-018-0045-8 (PMC5807510; doi:10.1038/s41525-018-0045-8)
Supplement: Supplementary file 1 — Supplementary Material 1 [file 41525_2018_45_MOESM1_ESM.docx]

**Supplementary Appendix**

**Rapid Whole Genome Sequencing for Etiologic Diagnosis of Critically Ill Infants: The NSIGHT1 Randomized Controlled Trial**

**Authors:**

Josh E. Petrikin, Julie A. Cakici, Michelle M. Clark, Laurel K. Willig, Nathaly M. Sweeney, Emily G. Farrow, Carol J. Saunders, Isabelle Thiffault, Neil A. Miller, Lee Zellmer, Suzanne M. Herd; Anne M. Holmes; Serge Batalov, Narayanan Veeraraghavan, Laurie D. Smith, David M. Dimmock, Steve Leeder, Stephen F. Kingsmore.

**Table of Contents:**

**Figure S1**………………………………………………………………………………………………………………………………………………Page 2

**Table S1**……………………………………………………………………………………………………………………………………………….Page 3

**Table S2**……………………………………………………………………………………………………………………………………………….Page 8

**Table S3**……………………………………………………………………………………………………………………………………………….Page 10

**Table S4**……………………………………………………………………………………………………………………………………………….Page 12

**Table S5**……………………………………………………………………………………………………………………………………………….Page 13

**Figure S1: Relative frequency distribution (%) of WGS nucleotide variant sizes for trios receiving rapid WGS.** Metrics include trios randomized to rapid WGS plus standard tests and trios randomized to standard tests only who were cross-overs to rapid WGS. Ins: nucleotide (nt) insertion. SNV: Single nucleotide variant. Del: nucleotide deletion. Del.21+: Deletions of >21 nt.

**
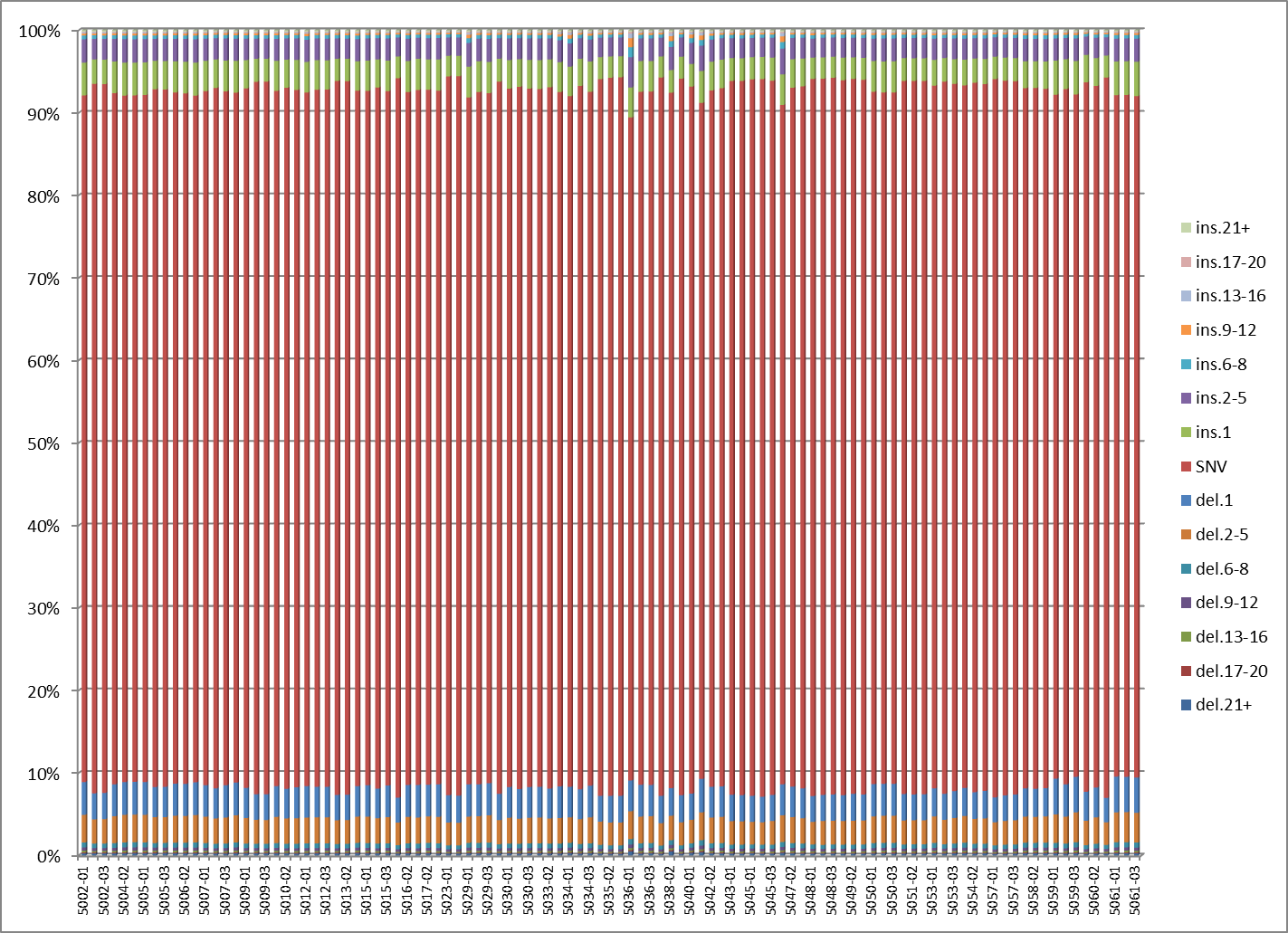
**

**Table S1: Clinical Features of Proband Infants who received rapid WGS or standard NGS diagnostic tests (panel tests, WES and WGS) in which primary analysis was guided by genes matching observed clinical features.** HPO: Human phenotype ontology. 5007, 5012, 5029, 5040 and 5053 were control subjects who crossed over to rWGS.

| **Patient ID** | **HPO Number** | **HPO Term** | **Number of HPO terms** | **Diagnosis (inc. possible Dx)** | **Phenomizer Rank of Diagnosis** | **Phenolyzer Rank of Diagnosis** |
| --- | --- | --- | --- | --- | --- | --- |
| 5002 | HP:0001622 | Premature birth | 10 | None |  |  |
|  | HP:0003084 | Fractures of the long bones |  |  |  |  |
|  | HP:0011682 | Perimembranous ventricular septal defect |  |  |  |  |
|  | HP:0001644 | Dilated cardiomyopathy |  |  |  |  |
|  | HP:0001667 | Right ventricular hypertrophy |  |  |  |  |
|  | HP:0008282 | Unconjugated hyperbilirubinemia |  |  |  |  |
|  | HP:0000325 | Triangular face |  |  |  |  |
|  | HP:0000368 | Low-set, posteriorly rotated ears |  |  |  |  |
|  | HP:0000609 | Optic nerve hypoplasia |  |  |  |  |
|  | HP:0006970 | Periventricular leukomalacia |  |  |  |  |
| 5004 | HP:0001998 | Neonatal hypoglycemia | 17 | 7p dup. | none | none |
|  | HP:0000842 | Hyperinsulinemia |  |  |  |  |
|  | HP:0000175 | Cleft palate |  |  |  |  |
|  | HP:0008527 | Congenital sensorineural hearing impairment |  |  |  |  |
|  | HP:0001873 | Thrombocytopenia |  |  |  |  |
|  | HP:0001319 | Neonatal hypotonia |  |  |  |  |
|  | HP:0009890 | High anterior hairline |  |  |  |  |
|  | HP:0000239 | Large fontanelles |  |  |  |  |
|  | HP:0000341 | Narrow forehead |  |  |  |  |
|  | HP:0000316 | Hypertelorism |  |  |  |  |
|  | HP:0012745 | Short palpebral fissure |  |  |  |  |
|  | HP:0009765 | Low hanging columella |  |  |  |  |
|  | HP:0000319 | Smooth philtrum |  |  |  |  |
|  | HP:0000160 | Narrow mouth |  |  |  |  |
|  | HP:0000347 | Micrognathia |  |  |  |  |
|  | HP:0004209 | Clinodactyly of the 5th finger |  |  |  |  |
|  | HP:0005617 | Bilateral camptodactyly |  |  |  |  |
| 5005 | HP:0001511 | Intrauterine growth retardation | 7 | None |  |  |
|  | HP:0002023 | Anal atresia |  |  |  |  |
|  | HP:0000061 | Ambiguous genitalia, female |  |  |  |  |
|  | HP:0002032 | Esophageal atresia |  |  |  |  |
|  | HP:0001562 | Oligohydramnios |  |  |  |  |
|  | HP:0100712 | Abnormality of the lumbar spine |  |  |  |  |
|  | HP:0001195 | Single umbilical artery |  |  |  |  |
| 5006 | HP:0000776 | Congenital diaphragmatic hernia | 7 | None |  |  |
|  | HP:0001539 | Omphalocele |  |  |  |  |
|  | HP:0001622 | Premature birth |  |  |  |  |
|  | HP:0010774 | Cor triatriatrum |  |  |  |  |
|  | HP:0011571 | Parachute mitral valve |  |  |  |  |
|  | HP:0005946 | Ventilator dependence with inability to wean |  |  |  |  |
|  | HP:0000879 | Short sternum |  |  |  |  |
| 5007 | HP:0000341 | Narrow forehead | 8 | *ALG1* | 8 | 157 |
|  | HP:0002015 | Dysphagia |  |  |  |  |
|  | HP:0002126 | Polymicrogyria |  |  |  |  |
|  | HP:0010307 | Stridor |  |  |  |  |
|  | HP:0011398 | Central hypotonia |  |  |  |  |
|  | HP:0012469 | Infantile spasms |  |  |  |  |
|  | HP:0001250 | Seizures |  |  |  |  |
|  | HP:0012345 | Abnormal glycosylation |  |  |  |  |
| 5008 | HP:0011675 | Arrhythmia | 9 | 8p23 del. | none | None |
|  | HP:0001674 | Complete atrioventricular canal defect |  |  |  |  |
|  | HP:0001059 | Pterygium |  |  |  |  |
|  | HP:0000041 | Chordee |  |  |  |  |
|  | HP:0000047 | Hypospadias |  |  |  |  |
|  | HP:0000154 | Wide mouth |  |  |  |  |
|  | HP:0000219 | Thin upper lip vermilion |  |  |  |  |
|  | HP:0012745 | Short palpebral fissure |  |  |  |  |
|  | HP:0001518 | Small for gestational age |  |  |  |  |
| 5009 | HP:0002023 | Anal atresia | 5 | None |  |  |
|  | HP:0002032 | Esophageal atresia |  |  |  |  |
|  | HP:0002575 | Tracheoesophageal fistula |  |  |  |  |
|  | HP:0001636 | Tetralogy of Fallot |  |  |  |  |
|  | HP:0002144 | Tethered cord |  |  |  |  |
| 5010 | HP:0001643 | Patent ductus arteriosus | 5 | None |  |  |
|  | HP:0011726 | Persistent fetal circulation |  |  |  |  |
|  | HP:0002092 | Pulmonary hypertension |  |  |  |  |
|  | HP:0000961 | Cyanosis |  |  |  |  |
|  | n.a. | Alveolar capillary dysplasia with misalignment of pulmonary veins | |  |  |  |
| 5012 | HP:0000023 | Inguinal hernia | 14 | None |  |  |
|  | HP:0000051 | Perineal hypospadias |  |  |  |  |
|  | HP:0000062 | Ambiguous genitalia |  |  |  |  |
|  | HP:0000384 | Preauricular skin tag |  |  |  |  |
|  | HP:0000520 | Proptosis |  |  |  |  |
|  | HP:0000609 | Optic nerve hypoplasia |  |  |  |  |
|  | HP:0000846 | Adrenal insufficiency |  |  |  |  |
|  | HP:0001508 | Failure to thrive |  |  |  |  |
|  | HP:0002093 | Respiratory insufficiency |  |  |  |  |
|  | HP:0006533 | Bronchodysplasia |  |  |  |  |
|  | HP:0008610 | Infantile sensorineural hearing impairment |  |  |  |  |
|  | HP:0008723 | Gonadal dysgenesis with female appearance, male |  |  |  |  |
|  | HP:0010944 | Abnormality of the renal pelvis |  |  |  |  |
|  | HP:0100779 | Urogenital sinus anomaly |  |  |  |  |
| 5013 | HP:0000368 | Low-set, posteriorly rotated ears | 14 | None |  |  |
|  | HP:0000308 | Microretrognathia |  |  |  |  |
|  | HP:0000474 | Thickened nuchal skin fold |  |  |  |  |
|  | HP:0010823 | Ridged cranial sutures |  |  |  |  |
|  | HP:0001290 | Generalized hypotonia |  |  |  |  |
|  | HP:0004532 | Sacral hypertrichosis |  |  |  |  |
|  | HP:0012368 | Flat face |  |  |  |  |
|  | HP:0000348 | High forehead |  |  |  |  |
|  | HP:0002553 | Highly arched eyebrow |  |  |  |  |
|  | HP:0010747 | Medial flaring of the eyebrow |  |  |  |  |
|  | HP:0000286 | Epicanthus |  |  |  |  |
|  | HP:0000162 | Glossoptosis |  |  |  |  |
|  | HP:0001601 | Laryngomalacia |  |  |  |  |
|  | HP:0002562 | Low-set nipples |  |  |  |  |
| 5014 | HP:0000857 | Neonatal diabetes mellitus | 1 | *ZFP57* | 16 | 1 |
| 5015 | HP:0009800 | Maternal diabetes | 3 | None |  |  |
|  | HP:0011679 | Tetralogy of Fallot with pulmonary stenosis |  |  |  |  |
|  | HP:0012020 | Right aortic arch |  |  |  |  |
| 5016 | HP:0000201 | Pierre-Robin sequence | 7 | None |  |  |
|  | HP:0000347 | Micrognathia |  |  |  |  |
|  | HP:0000185 | Cleft soft palate |  |  |  |  |
|  | HP:0000162 | Glossoptosis |  |  |  |  |
|  | HP:0010307 | Stridor |  |  |  |  |
|  | HP:0008591 | Congenital conductive hearing impairment |  |  |  |  |
|  | HP:0008527 | Congenital sensorineural hearing impairment |  |  |  |  |
| 5017 | HP:0011726 | Persistent fetal circulation | 9 | None |  |  |
|  | HP:0002092 | Pulmonary hypertension |  |  |  |  |
|  | HP:0002202 | Pleural effusion |  |  |  |  |
|  | HP:0001698 | Pericardial effusion |  |  |  |  |
|  | HP:0011649 | Patent ductus arteriosus after premature birth |  |  |  |  |
|  | HP:0001643 | Patent ductus arteriosus |  |  |  |  |
|  | HP:0010310 | Chylothorax |  |  |  |  |
|  | HP:0009800 | Maternal diabetes |  |  |  |  |
|  | HP:0005100 | Premature birth following premature rupture of fetal membranes |  |  |  |  |
| 5018 | HP:0001250 | Neonatal epilepsy | 1 | None |  |  |
| 5019 | HP:0001250 | Neonatal epilepsy | 2 | None |  |  |
|  | HP:0002133 | Status epilepticus |  |  |  |  |
| 5023 | HP:0000848 | Increased circulating renin level | 5 | *NR3C2* | 1 | 6 |
|  | HP:0000859 | Hyperaldosteronism |  |  |  |  |
|  | HP:0001530 | Mild postnatal growth retardation |  |  |  |  |
|  | HP:0002902 | Hyponatremia |  |  |  |  |
|  | HP:0008242 | Pseudohypoaldosteronism |  |  |  |  |
| 5029 | HP:0000057 | Clitoromegaly | 15 | None |  |  |
|  | HP:0000218 | High palate |  |  |  |  |
|  | HP:0000278 | Retrognathia |  |  |  |  |
|  | HP:0000294 | Low anterior hairline |  |  |  |  |
|  | HP:0000368 | Low-set, posteriorly rotated ears |  |  |  |  |
|  | HP:0000431 | Wide nasal bridge |  |  |  |  |
|  | HP:0001845 | Overlapping toe |  |  |  |  |
|  | HP:0001875 | Neutropenia |  |  |  |  |
|  | HP:0005280 | Depressed nasal bridge |  |  |  |  |
|  | HP:0005549 | Congenital neutropenia |  |  |  |  |
|  | HP:0009601 | Aplasia/Hypoplasia of the thumb |  |  |  |  |
|  | HP:0010557 | Overlapping fingers |  |  |  |  |
|  | HP:0010609 | Skin tags |  |  |  |  |
|  | HP:0010783 | Erythema |  |  |  |  |
|  | HP:0012385 | Camptodactyly |  |  |  |  |
| 5030 | HP:0002069 | Generalized tonic-clonic seizures | 2 | CHRNA4 | 28 | 106 |
|  | HP:0002919 | Ketonuria |  |  |  |  |
| 5033 | HP:0001692 | Primary atrial arrhythmia | 7 | SCN5A | 25 | 3 |
|  | HP:0005110 | Atrial fibrillation |  |  |  |  |
|  | HP:0004749 | Atrial flutter |  |  |  |  |
|  | HP:0001640 | Cardiomegaly |  |  |  |  |
|  | HP:0005162 | Left ventricular failure |  |  |  |  |
|  | HP:0001655 | Patent foramen ovale |  |  |  |  |
|  | HP:0002876 | Episodic tachypnea |  |  |  |  |
| 5034 | HP:0000776 | Congenital diaphragmatic hernia | 8 | None |  |  |
|  | HP:0009110 | Diaphragmatic eventration |  |  |  |  |
|  | HP:0011638 | Anomalous origin of left coronary artery from pulmonary artery |  |  |  |  |
|  | HP:0001680 | Coarctation of aorta |  |  |  |  |
|  | HP:0010773 | Partial anomalous pulmonary venous return |  |  |  |  |
|  | HP:0011626 | Scimitar anomaly |  |  |  |  |
|  | HP:0001684 | Secundum atrial septal defect |  |  |  |  |
|  | HP:0002089 | Pulmonary hypoplasia |  |  |  |  |
| 5035 | HP:0011451 | Congenital microcephaly | 1 | ASPM | 32 | none |
| 5036 | HP:0008872 | Feeding difficulties in infancy | 4 | PHOX2B | 1 | 19 |
|  | HP:0000975 | Hyperhidrosis |  |  |  |  |
|  | HP:0007110 | Central hypoventilation |  |  |  |  |
|  | HP:0002791 | Hypoventilation |  |  |  |  |
| 5038 | HP:0001696 | Situs inversus totalis | 1 | DNAH11 | 12 | 20 |
| 5040 | HP:0100492 | Joint contractures involving joints of feet | 10 | None |  |  |
|  | HP:0003273 | Hip contractures |  |  |  |  |
|  | HP:002804 | Congenital multiple arthrogryposis |  |  |  |  |
|  | HP:0001276 | Hypertonicity |  |  |  |  |
|  | HP:0001999 | Dysmorphic facies |  |  |  |  |
|  | HP:0011968 | Difficulty feeding |  |  |  |  |
|  | HP:0002104 | Apnea |  |  |  |  |
|  | HP:0012544 | Elevated aldolase |  |  |  |  |
|  | HP:0001274 | Agenesis of corpus callosum |  |  |  |  |
|  | HP:0200136 | Oropharyngeal dysphagia |  |  |  |  |
| 5042 | HP:0001319 | Neonatal hypotonia | 4 | PURA | 8 | none |
|  | HP:0006830 | Severe neonatal hypotonia in males |  |  |  |  |
|  | HP:0001336 | Myoclonic jerks |  |  |  |  |
|  | HP:0005949 | Apneic episodes in infancy |  |  |  |  |
| 5043 | HP:0000003 | Multicystic kidney dysplasia | 6 | None |  |  |
|  | HP:0000047 | Hypospadias |  |  |  |  |
|  | HP:0000126 | Hydronephrosis |  |  |  |  |
|  | HP:0001250 | Seizures |  |  |  |  |
|  | HP:0001622 | Premature birth |  |  |  |  |
|  | HP:0001643 | Patent ductus arteriosus |  |  |  |  |
| 5044 | HP:0001250 | Epilepsy | 1 | None |  |  |
| 5045 | HP:0002247 | Duodenal atresia | 6 | None |  |  |
|  | HP:0001636 | Tetralogy of Fallot |  |  |  |  |
|  | HP:0012541 | Cephalohematoma |  |  |  |  |
|  | HP:0008050 | Abnormality of the palpebral fissures |  |  |  |  |
|  | HP:0001631 | Atria septal defect |  |  |  |  |
|  | HP:0000598 | Abnormality of the ear |  |  |  |  |
| 5047 | HP:0000612 | Iris coloboma | 3 | None |  |  |
|  | HP:0001113 | Early cataracts |  |  |  |  |
|  | HP:0100259 | Postaxial polydactyly |  |  |  |  |
| 5048 | HP:0001250 | Seizures | 1 | KCNT1 | 37 | 265 |
| 5049 | HP:0001433 | Hepatosplenomegaly | 8 | None |  |  |
|  | HP:0001905 | Congenital thrombocytopenia |  |  |  |  |
|  | HP:0004810 | Congenital hypoplastic anemia |  |  |  |  |
|  | HP:0000952 | Jaundice |  |  |  |  |
|  | HP:0003281 | Increased serum ferritin |  |  |  |  |
|  | HP:0011117 | Abnormality of interleukin secretion |  |  |  |  |
|  | HP:0000967 | Petechiae |  |  |  |  |
|  | HP:0001290 | Generalized hypotonia |  |  |  |  |
| 5050 | HP:0002089 | Pulmonary hypoplasia | 2 | None |  |  |
|  | HP:0006530 | Interstitial lung disease |  |  |  |  |
| 5051 | HP:0001791 | Fetal ascites | 3 | PIEZO1 | 1774 | none |
|  | HP:0002908 | Conjugated hyperbilirubinemia |  |  |  |  |
|  | HP:0002910 | Elevated hepatic transaminases |  |  |  |  |
| 5053 | HP:0006830 | Severe neonatal hypotonia in males | 6 | MTM1 | 757 | 193 |
|  | HP:0000028 | Cryptorchidism |  |  |  |  |
|  | HP:0000341 | Narrow forehead |  |  |  |  |
|  | HP:0008559 | Hypoplastic superior helix |  |  |  |  |
|  | HP:0004691 | 2-3 toe syndactyly |  |  |  |  |
|  | HP:0010511 | Long toe |  |  |  |  |
| 5054 | HP:0010851 | EEG with burst suppression | 2 | None |  |  |
|  | HP:0011649 | Patent ductus arteriosus after premature birth |  |  |  |  |
| 5057 | HP:0000316 | Hypertelorism | 6 | SOS1 | 3 | 14 |
|  | HP:0001561 | Polyhydramnios |  |  |  |  |
|  | HP:0000474 | Thickened nuchal skin fold |  |  |  |  |
|  | HP:0001631 | Atrial septal defect |  |  |  |  |
|  | HP:0005176 | Dysplastic aortic valve |  |  |  |  |
|  | HP:0005164 | Dysplastic pulmonary valve |  |  |  |  |
| 5058 | HP:0001999 | Dysmorphic features | 4 | None |  |  |
|  | HP:0008947 | Hypotonia |  |  |  |  |
|  | HP:0002126 | Polymicrogyria |  |  |  |  |
|  | HP:0001629 | Small VSD |  |  |  |  |
| 5059 | HP:0004383 | Hypoplastic Left Heart Syndrome | 10 | ARID1A | 24 | 81 |
|  | HP:0002144 | Tethered cord |  |  |  |  |
|  | HP:0000028 | Undescended testes |  |  |  |  |
|  | HP:0004736 | Crossed Fused renal ectopia |  |  |  |  |
|  | HP:0002119 | Enlarged Cerebral Ventricles |  |  |  |  |
|  | HP:0011217 | Abnormal shape of posterior cranium |  |  |  |  |
|  | HP:0001195 | Single umbilical artery |  |  |  |  |
|  | HP:0003097 | Short femurs |  |  |  |  |
|  | HP:0000315 | Abnormality of orbital region |  |  |  |  |
|  | HP:0001188 | Clenched hands |  |  |  |  |
| 5060 | HP:0004378 | Abnormality of the anus | 3 | None |  |  |
|  | HP:0001058 | Poor wound healing |  |  |  |  |
|  | HP:0002144 | Tethered cord |  |  |  |  |
| 5061 | HP:0011398 | Central hypotonia | 5 | GLRA1 | 4 | 15 |
|  | HP:0009062 | Infantile axial hypotonia |  |  |  |  |
|  | HP:0007033 | Cerebellar dysplasia |  |  |  |  |
|  | HP:0001263 | Global developmental delay |  |  |  |  |
|  | HP:0002267 | Exaggerated startle response |  |  |  |  |
| 5062 | HP:0001647 | Bicuspid aortic valve | 6 | RYR1 | 270 | 7016 |
|  | HP:0001531 | Failure to thrive in infancy |  |  |  |  |
|  | HP:0008947 | Infantile muscular hypotonia |  |  |  |  |
|  | HP:0001974 | Leukocytosis |  |  |  |  |
|  | HP:0003458 | Myopathy |  |  |  |  |
|  | HP:0002747 | Respiratory insufficiency due to muscle weakness |  |  |  |  |
| **Average** | |  | 5.9 |  | 188 | 607 |
| **% Ranked** | |  |  |  | 88.9% | 72.2% |

**Table S2: Nucleotide variant metrics for trios receiving rapid WGS.** Metrics include trios randomized to rapid WGS plus standard tests and trios randomized to standard tests only who were cross-overs to rapid WGS. Ins: nucleotide (nt) insertion. SNV: Single nucleotide variant. Del: nucleotide deletion. Del.21+: Deletions of >21 nt.

| **Sample ID** | **del.21+** | **del.17-20** | **del.13-16** | **del.9-12** | **del.6-8** | **del.2-5** | **del.1** | **SNV** | **ins.1** | **ins.2-5** | **ins.6-8** | **ins.9-12** | **ins.13-16** | **ins.17-20** | **ins.21+** | **Del** | **SNV** | **ins** | **Total** |
| --- | --- | --- | --- | --- | --- | --- | --- | --- | --- | --- | --- | --- | --- | --- | --- | --- | --- | --- | --- |
| **5002-01** | 13195 | 5855 | 10231 | 19377 | 31108 | 169845 | 201685 | 4217405 | 201574 | 143980 | 22794 | 12835 | 6555 | 4012 | 8734 | 451296 | 4217405 | 400484 | 5069185 |
| **5002-02** | 12249 | 5235 | 8751 | 16671 | 26860 | 142619 | 153764 | 4181380 | 143539 | 122270 | 20388 | 11604 | 5992 | 3697 | 8651 | 366149 | 4181380 | 316141 | 4863670 |
| **5002-03** | 12733 | 5103 | 8703 | 16632 | 26629 | 142639 | 153766 | 4147955 | 142805 | 121731 | 20344 | 11525 | 5926 | 3716 | 8852 | 366205 | 4147955 | 314899 | 4829059 |
| **5004-01** | 12590 | 5616 | 9891 | 18661 | 29932 | 165813 | 198183 | 4277871 | 195557 | 140660 | 21928 | 12494 | 6221 | 3906 | 8510 | 440686 | 4277871 | 389276 | 5107833 |
| **5004-02** | 13543 | 6013 | 10364 | 19791 | 31649 | 173627 | 207368 | 4320228 | 206912 | 147929 | 23039 | 13233 | 6689 | 4100 | 8915 | 462355 | 4320228 | 410817 | 5193400 |
| **5004-03** | 13613 | 5945 | 10309 | 19528 | 31118 | 169386 | 200327 | 4204798 | 200605 | 143075 | 22624 | 12980 | 6568 | 3941 | 9063 | 450226 | 4204798 | 398856 | 5053880 |
| **5005-01** | 13463 | 5912 | 10397 | 19332 | 31160 | 170100 | 200877 | 4231791 | 200849 | 143873 | 22288 | 12977 | 6480 | 4031 | 8691 | 451241 | 4231791 | 399189 | 5082221 |
| **5005-02** | 13302 | 5496 | 9612 | 17694 | 28514 | 155810 | 181451 | 4200586 | 172048 | 132312 | 20849 | 11855 | 6150 | 3851 | 8945 | 411879 | 4200586 | 356010 | 4968475 |
| **5005-03** | 13692 | 5500 | 9530 | 17889 | 28384 | 155863 | 181619 | 4190835 | 172325 | 132321 | 21102 | 12034 | 6253 | 3817 | 9113 | 412477 | 4190835 | 356965 | 4960277 |
| **5006-01** | 12872 | 5733 | 9864 | 18684 | 29842 | 162989 | 194114 | 4185150 | 187716 | 136445 | 21296 | 12154 | 6300 | 3875 | 8181 | 434098 | 4185150 | 375967 | 4995215 |
| **5006-02** | 12622 | 5712 | 9849 | 18649 | 29954 | 163051 | 194535 | 4190315 | 191764 | 138674 | 21736 | 12298 | 6371 | 3904 | 8418 | 434372 | 4190315 | 383165 | 5007852 |
| **5006-03** | 12824 | 5776 | 10123 | 19262 | 30847 | 167603 | 199794 | 4191546 | 200388 | 143417 | 22347 | 12869 | 6597 | 3862 | 8891 | 446229 | 4191546 | 398371 | 5036146 |
| **5007-01** | 12653 | 5488 | 9526 | 18118 | 29116 | 159572 | 190442 | 4208404 | 183579 | 134571 | 21027 | 11948 | 6076 | 3756 | 8043 | 424915 | 4208404 | 369000 | 5002319 |
| **5007-02** | 11595 | 5100 | 8819 | 16941 | 27693 | 151322 | 181152 | 4205551 | 170751 | 127124 | 20349 | 11251 | 5843 | 3561 | 7038 | 402622 | 4205551 | 345917 | 4954090 |
| **5007-03** | 11702 | 5246 | 9306 | 17595 | 28312 | 157851 | 195453 | 4205968 | 187040 | 133965 | 20750 | 11764 | 5975 | 3641 | 7163 | 425465 | 4205968 | 370298 | 5001731 |
| **5008-01** | 15393 | 6749 | 11778 | 22230 | 35009 | 192163 | 231780 | 4879650 | 225694 | 157250 | 24419 | 13780 | 7020 | 4303 | 9417 | 515102 | 4879650 | 441883 | 5836635 |
| **5009-01** | 12038 | 5201 | 9049 | 17050 | 27308 | 150770 | 178446 | 4148016 | 167944 | 127380 | 20152 | 11571 | 5896 | 3679 | 7789 | 399862 | 4148016 | 344411 | 4892289 |
| **5009-02** | 12542 | 5107 | 8519 | 16129 | 26033 | 140762 | 150296 | 4186452 | 134573 | 119441 | 19782 | 11152 | 5752 | 3527 | 8349 | 359388 | 4186452 | 302576 | 4848416 |
| **5009-03** | 12377 | 4910 | 8522 | 15974 | 25542 | 139242 | 150021 | 4156851 | 132576 | 118276 | 19548 | 11202 | 5575 | 3488 | 8426 | 356588 | 4156851 | 299091 | 4812530 |
| **5010-01** | 12731 | 5371 | 9454 | 17685 | 28491 | 157491 | 186268 | 4181905 | 180237 | 133006 | 21063 | 11828 | 6049 | 3763 | 8394 | 417491 | 4181905 | 364340 | 4963736 |
| **5010-02** | 11806 | 5050 | 8807 | 16534 | 26705 | 148973 | 174530 | 4126953 | 165224 | 125286 | 19704 | 11101 | 5758 | 3605 | 7537 | 392405 | 4126953 | 338215 | 4857573 |
| **5010-03** | 11729 | 5001 | 8853 | 16485 | 26517 | 151060 | 183575 | 4124311 | 177907 | 128200 | 19804 | 11146 | 5762 | 3522 | 7456 | 403220 | 4124311 | 353797 | 4881328 |
| **5012-01** | 12192 | 5163 | 9025 | 17425 | 28245 | 154596 | 189950 | 4164627 | 183334 | 132205 | 22747 | 14873 | 8000 | 4448 | 7630 | 416596 | 4164627 | 373237 | 4954460 |
| **5012-02** | 12439 | 5437 | 9270 | 17708 | 28448 | 157616 | 186669 | 4234573 | 180089 | 132305 | 20735 | 11763 | 6075 | 3543 | 7684 | 417587 | 4234573 | 362194 | 5014354 |
| **5012-03** | 12277 | 5321 | 9119 | 17272 | 27983 | 154938 | 182673 | 4156262 | 173993 | 130216 | 20474 | 11482 | 5976 | 3724 | 7625 | 409583 | 4156262 | 353490 | 4919335 |
| **5013-01** | 12276 | 4913 | 8318 | 15785 | 25647 | 138861 | 147304 | 4173716 | 130446 | 118066 | 19801 | 11092 | 5630 | 3378 | 8292 | 353104 | 4173716 | 296705 | 4823525 |
| **5013-02** | 12423 | 4970 | 8236 | 15937 | 25916 | 139102 | 147760 | 4174377 | 130239 | 118846 | 19907 | 11145 | 5689 | 3443 | 8249 | 354344 | 4174377 | 297518 | 4826239 |
| **5013-03** | 13536 | 5593 | 9623 | 18077 | 29049 | 157323 | 183401 | 4186188 | 176551 | 134420 | 21453 | 12108 | 6265 | 3794 | 9161 | 416602 | 4186188 | 363752 | 4966542 |
| **5015-01** | 12632 | 5407 | 9484 | 17765 | 28629 | 158887 | 189172 | 4178661 | 182405 | 133106 | 20560 | 11657 | 5885 | 3672 | 7875 | 421976 | 4178661 | 365160 | 4965797 |
| **5015-02** | 11892 | 5066 | 9020 | 16817 | 27316 | 151254 | 177302 | 4182756 | 167673 | 125946 | 19861 | 11082 | 5688 | 3616 | 7328 | 398667 | 4182756 | 341194 | 4922617 |
| **5015-03** | 12257 | 5454 | 9264 | 17542 | 28168 | 157265 | 188804 | 4161562 | 182901 | 132705 | 20398 | 11443 | 5978 | 3610 | 7619 | 418754 | 4161562 | 364654 | 4944970 |
| **5016-01** | 10175 | 4282 | 7388 | 13964 | 22803 | 131077 | 143087 | 4149480 | 124120 | 110065 | 17643 | 9823 | 4858 | 3023 | 6469 | 332776 | 4149480 | 276001 | 4758257 |
| **5016-02** | 12152 | 5461 | 9563 | 17819 | 28873 | 160502 | 193328 | 4211864 | 188665 | 136719 | 21291 | 12103 | 5979 | 3734 | 7535 | 427698 | 4211864 | 376026 | 5015588 |
| **5017-01** | 12806 | 6246 | 10722 | 20433 | 33027 | 184087 | 230373 | 4903526 | 220134 | 148523 | 22590 | 12651 | 6416 | 3918 | 7302 | 497694 | 4903526 | 421534 | 5822754 |
| **5017-02** | 14878 | 6664 | 11497 | 21682 | 34646 | 192171 | 228509 | 5010056 | 218800 | 153865 | 23680 | 13424 | 6926 | 4180 | 8891 | 510047 | 5010056 | 429766 | 5949869 |
| **5017-03** | 13571 | 6315 | 11078 | 21134 | 33206 | 187169 | 231126 | 4907303 | 222133 | 151643 | 22718 | 13035 | 6626 | 3899 | 7829 | 503599 | 4907303 | 427883 | 5838785 |
| **5023-01** | 10902 | 4397 | 7449 | 14265 | 23453 | 137288 | 164455 | 4329362 | 123350 | 111144 | 18099 | 10106 | 5060 | 3035 | 6725 | 362209 | 4329362 | 277519 | 4969090 |
| **5023-02** | 10940 | 4354 | 7374 | 14359 | 23284 | 135770 | 162620 | 4310988 | 121876 | 110730 | 17976 | 10132 | 5093 | 3044 | 6790 | 358701 | 4310988 | 275641 | 4945330 |
| **5029-01** | 12031 | 5021 | 9130 | 17340 | 27573 | 149506 | 183967 | 3907779 | 175529 | 135181 | 26535 | 19009 | 10892 | 6036 | 9100 | 404568 | 3907779 | 382282 | 4694629 |
| **5029-02** | 11469 | 5104 | 9120 | 17572 | 27920 | 153454 | 183004 | 3965442 | 176174 | 130282 | 20398 | 11558 | 5804 | 3336 | 6453 | 407643 | 3965442 | 354005 | 4727090 |
| **5029-03** | 12077 | 5253 | 9248 | 17515 | 28405 | 155955 | 183397 | 3937936 | 177605 | 132832 | 20699 | 11797 | 5712 | 3339 | 7005 | 411850 | 3937936 | 358989 | 4708775 |
| **5029-04** | 10877 | 4342 | 7734 | 14464 | 23587 | 132037 | 143637 | 3898247 | 125720 | 112170 | 18285 | 10329 | 5082 | 3026 | 6736 | 336678 | 3898247 | 281348 | 4516273 |
| **5030-01** | 12108 | 5288 | 9013 | 17180 | 27857 | 154980 | 185367 | 4198992 | 172153 | 129579 | 20566 | 11672 | 5884 | 3667 | 7544 | 411793 | 4198992 | 351065 | 4961850 |
| **5030-02** | 11358 | 5049 | 8654 | 16701 | 27058 | 149094 | 176464 | 4178286 | 163538 | 124800 | 19942 | 11252 | 5659 | 3572 | 7011 | 394378 | 4178286 | 335774 | 4908438 |
| **5030-03** | 12277 | 5417 | 9112 | 17447 | 28022 | 155282 | 185152 | 4219073 | 172756 | 129898 | 20595 | 11519 | 5920 | 3613 | 7605 | 412709 | 4219073 | 351906 | 4983688 |
| **5033-01** | 12211 | 5303 | 9290 | 17451 | 28169 | 154552 | 184149 | 4186273 | 173327 | 129615 | 20559 | 11646 | 5919 | 3679 | 7599 | 411125 | 4186273 | 352344 | 4949742 |
| **5033-02** | 11775 | 5104 | 8818 | 16896 | 27529 | 150723 | 177721 | 4185786 | 166069 | 126541 | 20372 | 11225 | 5772 | 3608 | 7135 | 398566 | 4185786 | 340722 | 4925074 |
| **5033-03** | 11609 | 5220 | 9119 | 17396 | 28090 | 153217 | 190753 | 4182684 | 180301 | 131598 | 22979 | 14939 | 8534 | 5196 | 8882 | 415404 | 4182684 | 372429 | 4970517 |
| **5034-01** | 13634 | 5491 | 9765 | 18420 | 29592 | 156215 | 185481 | 4219372 | 179710 | 140947 | 29705 | 22643 | 12498 | 6645 | 9331 | 418598 | 4219372 | 401479 | 5039449 |
| **5034-02** | 11258 | 4869 | 8517 | 16203 | 26383 | 148178 | 177495 | 4176405 | 160889 | 123132 | 19654 | 11017 | 5640 | 3420 | 6881 | 392903 | 4176405 | 330633 | 4899941 |
| **5034-03** | 12233 | 5328 | 9503 | 17765 | 28879 | 157843 | 192021 | 4215121 | 184156 | 134557 | 22562 | 13781 | 7315 | 4346 | 7796 | 423572 | 4215121 | 374513 | 5013206 |
| **5035-01** | 10868 | 4443 | 7593 | 14617 | 23727 | 135685 | 150050 | 4175681 | 128126 | 113447 | 18367 | 10287 | 5100 | 3098 | 6966 | 346983 | 4175681 | 285391 | 4808055 |
| **5035-02** | 10031 | 4297 | 7293 | 14046 | 22899 | 134651 | 156092 | 4214594 | 124784 | 110805 | 18000 | 10088 | 5134 | 3072 | 6638 | 349309 | 4214594 | 278521 | 4842424 |
| **5035-03** | 10329 | 4349 | 7410 | 14160 | 22974 | 135483 | 157826 | 4247794 | 123997 | 110604 | 17971 | 10211 | 5148 | 3130 | 6580 | 352531 | 4247794 | 277641 | 4877966 |
| **5036-01** | 25342 | 7315 | 12107 | 22695 | 33626 | 170708 | 189956 | 4088182 | 184784 | 186277 | 61640 | 55845 | 27113 | 11086 | 10414 | 461749 | 4088182 | 537159 | 5087090 |
| **5036-02** | 10966 | 5071 | 9149 | 17423 | 27995 | 154817 | 184594 | 4024174 | 179308 | 131255 | 20526 | 11618 | 5588 | 3315 | 6378 | 410015 | 4024174 | 357988 | 4792177 |
| **5036-03** | 11588 | 5207 | 9231 | 17644 | 28293 | 156002 | 182258 | 4033208 | 176914 | 131966 | 20416 | 11259 | 5674 | 3367 | 6676 | 410223 | 4033208 | 356272 | 4799703 |
| **5038-01** | 9499 | 3998 | 6994 | 13499 | 21924 | 125557 | 155630 | 4063872 | 119446 | 105958 | 17775 | 10161 | 5179 | 2978 | 5789 | 337101 | 4063872 | 267286 | 4668259 |
| **5038-02** | 24188 | 6470 | 10268 | 18583 | 27872 | 140527 | 157375 | 3991250 | 129691 | 132462 | 33440 | 29173 | 16123 | 7885 | 9371 | 385283 | 3991250 | 358145 | 4734678 |
| **5038-03** | 9335 | 4077 | 7072 | 13757 | 22492 | 128823 | 151520 | 4013965 | 122444 | 107769 | 17414 | 9736 | 4866 | 2902 | 5821 | 337076 | 4013965 | 270952 | 4621993 |
| **5040-01** | 14938 | 5140 | 8752 | 16437 | 25882 | 136912 | 154702 | 4141109 | 131836 | 124087 | 27327 | 21256 | 11275 | 5687 | 8025 | 362763 | 4141109 | 329493 | 4833365 |
| **5042-01** | 21994 | 7796 | 13001 | 24617 | 37650 | 193272 | 230651 | 4676115 | 220345 | 174906 | 39528 | 31429 | 17048 | 8282 | 10580 | 528981 | 4676115 | 502118 | 5707214 |
| **5042-02** | 11481 | 5061 | 8870 | 17142 | 27322 | 148592 | 177369 | 4024578 | 166216 | 127439 | 22124 | 13970 | 7122 | 4039 | 6616 | 395837 | 4024578 | 347526 | 4767941 |
| **5042-03** | 13793 | 6191 | 10786 | 20733 | 33057 | 179817 | 209189 | 4784930 | 196388 | 146807 | 23086 | 12940 | 6453 | 3850 | 7692 | 473566 | 4784930 | 397216 | 5655712 |
| **5043-01** | 10300 | 4357 | 7614 | 14609 | 23680 | 132337 | 150323 | 4035480 | 129783 | 112153 | 18995 | 10976 | 5490 | 3237 | 6487 | 343220 | 4035480 | 287121 | 4665821 |
| **5043-02** | 10378 | 4377 | 7645 | 14650 | 23557 | 131910 | 148414 | 4035073 | 127434 | 112208 | 19290 | 11368 | 5839 | 3300 | 6452 | 340931 | 4035073 | 285891 | 4661895 |
| **5045-01** | 10797 | 4228 | 7295 | 14076 | 22497 | 121369 | 139500 | 3854068 | 119717 | 102751 | 17609 | 10082 | 5153 | 2790 | 5934 | 319762 | 3854068 | 264036 | 4437866 |
| **5045-02** | 10232 | 4159 | 7142 | 13856 | 22311 | 120865 | 137113 | 3867055 | 119184 | 102874 | 17273 | 9809 | 4922 | 2788 | 5811 | 315678 | 3867055 | 262661 | 4445394 |
| **5045-03** | 9884 | 4401 | 7585 | 14655 | 23775 | 132094 | 146198 | 4001449 | 129117 | 111383 | 17997 | 10089 | 4863 | 2874 | 6269 | 338592 | 4001449 | 282592 | 4622633 |
| **5047-01** | 17454 | 6943 | 12083 | 23040 | 36040 | 190568 | 221703 | 4847274 | 216935 | 184132 | 47744 | 40396 | 21870 | 10044 | 11271 | 507831 | 4847274 | 532392 | 5887497 |
| **5047-02** | 13711 | 6177 | 10803 | 20633 | 32862 | 179712 | 209428 | 4811241 | 196702 | 146129 | 22831 | 12599 | 6285 | 3742 | 7284 | 473326 | 4811241 | 395572 | 5680139 |
| **5047-03** | 12763 | 6009 | 10384 | 19657 | 31896 | 174547 | 203917 | 4807757 | 189875 | 142114 | 22551 | 12328 | 6204 | 3629 | 7133 | 459173 | 4807757 | 383834 | 5650764 |
| **5048-01** | 12743 | 5077 | 8661 | 16721 | 26642 | 146028 | 166180 | 4626544 | 137747 | 122445 | 21927 | 13917 | 7108 | 4008 | 6999 | 382052 | 4626544 | 314151 | 5322747 |
| **5048-02** | 10174 | 4282 | 7447 | 14113 | 23170 | 133988 | 146855 | 4022859 | 119389 | 110404 | 17874 | 9990 | 4824 | 2892 | 6388 | 340029 | 4022859 | 271761 | 4634649 |
| **5048-03** | 12738 | 5633 | 9250 | 17410 | 28104 | 160423 | 177234 | 4824875 | 142413 | 128308 | 20954 | 11573 | 5734 | 3449 | 7534 | 410792 | 4824875 | 319965 | 5555632 |
| **5049-01** | 10118 | 4330 | 7600 | 14689 | 23919 | 132494 | 144947 | 4018616 | 126411 | 111614 | 18223 | 10259 | 4989 | 2952 | 6308 | 338097 | 4018616 | 280756 | 4637469 |
| **5049-02** | 10397 | 4400 | 7466 | 14482 | 23456 | 135167 | 152753 | 4035798 | 121258 | 111288 | 18099 | 10029 | 4907 | 2959 | 6499 | 348121 | 4035798 | 275039 | 4658958 |
| **5049-03** | 10713 | 4448 | 7687 | 14722 | 23722 | 134127 | 146846 | 4007455 | 123305 | 111743 | 18180 | 10249 | 4959 | 3036 | 6630 | 342265 | 4007455 | 278102 | 4627822 |
| **5050-01** | 11160 | 5220 | 9037 | 17565 | 28361 | 156087 | 188446 | 4041603 | 179175 | 131005 | 20851 | 11807 | 5805 | 3509 | 6383 | 415876 | 4041603 | 358535 | 4816014 |
| **5050-02** | 11748 | 5323 | 9351 | 17872 | 28738 | 157714 | 185737 | 4024470 | 180641 | 134058 | 20716 | 11510 | 5653 | 3349 | 6618 | 416483 | 4024470 | 362545 | 4803498 |
| **5050-03** | 11501 | 5334 | 9282 | 17710 | 28465 | 157425 | 185798 | 4012820 | 180836 | 132730 | 20741 | 11389 | 5530 | 3348 | 6485 | 415515 | 4012820 | 361059 | 4789394 |
| **5051-01** | 10605 | 4533 | 7690 | 14897 | 23967 | 135332 | 149194 | 4015342 | 129018 | 113355 | 18279 | 10199 | 4926 | 2971 | 6700 | 346218 | 4015342 | 285448 | 4647008 |
| **5051-02** | 10921 | 4671 | 7883 | 14903 | 24353 | 135855 | 145953 | 4028117 | 127948 | 114454 | 18445 | 10262 | 5070 | 3079 | 6963 | 344539 | 4028117 | 286221 | 4658877 |
| **5051-03** | 10884 | 4562 | 7812 | 15051 | 24059 | 134827 | 146157 | 4000661 | 127436 | 113476 | 18372 | 10284 | 4977 | 3010 | 6952 | 343352 | 4000661 | 284507 | 4628520 |
| **5053-01** | 12397 | 5267 | 9057 | 17155 | 28127 | 152891 | 161555 | 4049693 | 148937 | 122529 | 19604 | 11190 | 5617 | 3480 | 7839 | 386449 | 4049693 | 319196 | 4755338 |
| **5053-02** | 11067 | 4788 | 8312 | 15738 | 25890 | 143376 | 153396 | 4194664 | 138844 | 117547 | 18795 | 10996 | 5448 | 3284 | 7158 | 362567 | 4194664 | 302072 | 4859303 |
| **5053-03** | 11534 | 4844 | 8400 | 16041 | 26039 | 143912 | 152474 | 3988647 | 139557 | 117503 | 19006 | 10738 | 5407 | 3246 | 7328 | 363244 | 3988647 | 302785 | 4654676 |
| **5054-01** | 12513 | 5277 | 9169 | 17327 | 28518 | 153369 | 161583 | 4040557 | 148208 | 121535 | 19531 | 11169 | 5732 | 3483 | 7948 | 387756 | 4040557 | 317606 | 4745919 |
| **5054-02** | 11377 | 4915 | 8476 | 16087 | 26150 | 144720 | 154369 | 4110172 | 140641 | 117790 | 19200 | 10872 | 5521 | 3345 | 7241 | 366094 | 4110172 | 304610 | 4780876 |
| **5054-03** | 11370 | 4889 | 8348 | 15925 | 26153 | 144703 | 157072 | 4036834 | 143368 | 118126 | 19159 | 10929 | 5476 | 3348 | 7243 | 368460 | 4036834 | 307649 | 4712943 |
| **5057-01** | 9059 | 4075 | 7102 | 13676 | 22819 | 131881 | 142487 | 4105171 | 128144 | 110030 | 17400 | 9921 | 4714 | 2874 | 5758 | 331099 | 4105171 | 278841 | 4715111 |
| **5057-02** | 9859 | 4358 | 7559 | 14704 | 24101 | 136448 | 145525 | 4078166 | 131735 | 113728 | 18288 | 10213 | 4864 | 3014 | 6286 | 342554 | 4078166 | 288128 | 4708848 |
| **5057-03** | 10266 | 4461 | 7721 | 15011 | 24275 | 137202 | 145446 | 4037647 | 131422 | 113816 | 18347 | 10454 | 5081 | 3105 | 6486 | 344382 | 4037647 | 288711 | 4670740 |
| **5058-01** | 11995 | 5315 | 9321 | 18110 | 28845 | 150975 | 162523 | 4047806 | 155366 | 129183 | 21083 | 11953 | 5994 | 3658 | 7473 | 387084 | 4047806 | 334710 | 4769600 |
| **5058-02** | 11920 | 5180 | 9200 | 17941 | 28611 | 150740 | 161438 | 4058115 | 155662 | 129000 | 21269 | 11826 | 5971 | 3541 | 7296 | 385030 | 4058115 | 334565 | 4777710 |
| **5058-03** | 12087 | 5370 | 9444 | 18169 | 29244 | 152839 | 165008 | 4082866 | 159746 | 131162 | 21679 | 12369 | 6170 | 3677 | 7486 | 392161 | 4082866 | 342289 | 4817316 |
| **5059-01** | 12255 | 5462 | 9989 | 18682 | 30155 | 173672 | 219088 | 4182567 | 208846 | 136068 | 21005 | 12037 | 5912 | 3541 | 6708 | 469303 | 4182567 | 394117 | 5045987 |
| **5059-02** | 11798 | 5224 | 9445 | 18025 | 29314 | 166749 | 201399 | 4325689 | 186393 | 131974 | 20385 | 11658 | 5797 | 3454 | 6642 | 441954 | 4325689 | 366303 | 5133946 |
| **5059-03** | 13128 | 5790 | 10299 | 19562 | 31363 | 183221 | 216181 | 4168324 | 204249 | 137243 | 21277 | 12124 | 6060 | 3593 | 7303 | 479544 | 4168324 | 391849 | 5039717 |
| **5060-01** | 11050 | 5351 | 9282 | 18000 | 29942 | 172453 | 207317 | 5048712 | 194675 | 131214 | 19634 | 10942 | 5332 | 3153 | 5787 | 453395 | 5048712 | 370737 | 5872844 |
| **5060-02** | 11823 | 5368 | 9279 | 18153 | 29490 | 167551 | 190699 | 4461131 | 176791 | 129597 | 20029 | 11387 | 5721 | 3464 | 6750 | 432363 | 4461131 | 353739 | 5247233 |
| **5060-03** | 11776 | 5086 | 8656 | 16606 | 27386 | 148840 | 160916 | 4758988 | 145838 | 119910 | 19448 | 11107 | 5569 | 3372 | 7265 | 379266 | 4758988 | 312509 | 5450763 |
| **5061-01** | 12766 | 5652 | 9912 | 18810 | 30661 | 177847 | 212198 | 4041053 | 200294 | 135590 | 20925 | 11813 | 5944 | 3551 | 6971 | 467846 | 4041053 | 385088 | 4893987 |
| **5061-02** | 12882 | 5671 | 10110 | 19139 | 31192 | 180927 | 212578 | 4104114 | 202649 | 136878 | 21029 | 11963 | 5975 | 3559 | 7058 | 472499 | 4104114 | 389111 | 4965724 |
| **5061-03** | 12143 | 5442 | 9684 | 18527 | 30172 | 175834 | 208808 | 4024373 | 203375 | 136131 | 20860 | 11981 | 5914 | 3472 | 6920 | 460610 | 4024373 | 388653 | 4873636 |

**Table S3. Standard (Std.) clinical genetic tests ordered in enrolled probands.** FISH: Fluorescence in situ hybridization. CMA: Chromsomal microarray. CNV: Copy number variation. CSF: cerebrospinal fluid. Ctrl: Control. Del/Dup: Deletion/Duplication. DEB: Cytogenetic diepoxybutane clastogen assay for Fanconi anemia. F5L/F2: Molecular test for two variants associated with thrombophilia (Factor 5 Leiden (1691G>A) and prothrombin (F2 20210G>A)). NGS: Targeted next generation sequencing panels. SMA: spinal muscular atrophy exon 7 deletion analysis. WES: Whole exome sequencing. WGS: whole genome sequencing. *Del/Dup and CNV testing was performed as part of some tests; these tests were counted once. All infants also received at least two Missouri state newborn screens, which were not included in the number of standard tests ordered. *Controls 5007, 5012, 5029, 5040 and 5053 were crossed over to rWGS.

| **Patient ID** | **Study Arm** | **Number of Std. Tests Ordered** | **Chromosomal Testing (Karyotype, High Resolution, FISH)** | **CMA** | **Clinical**  **Targeted NGS Panel, WES, WGS** | **Methyl-ation** | **CNV*** | **Del/ Dup*** | **Other Tests** | **Other Test Description** |
| --- | --- | --- | --- | --- | --- | --- | --- | --- | --- | --- |
| **5000** | Ctrl | 3 | 1 | 1 | 1 |  |  |  |  |  |
| **5001** | Ctrl | 6 | 1 | 1 | 3 |  |  | 1 | 1 | DEB |
| **5002** | Case | 1 |  |  |  | 1 |  |  |  |  |
| **5003** | Ctrl | 10 |  |  |  |  |  |  | 10 | Acylcarnitine; Urine Organic Acids; Lactate; CSF Glycine; CSF Amino Acids; Ammonia; Serum Amino Acids; Pippa Colic Lysomal; Urine Pippa; Pyruvate; Urine Alpha; Pippa Acid Serum |
| **5004** | Case | 3 | 1 | 1 | 2 |  |  |  |  |  |
| **5005** | Case | 4 | 2 | 1 | 1 |  | 1 | 1 |  |  |
| **5006** | Case | 1 |  | 1 |  |  |  |  |  |  |
| **5007** | Ctrl * | 3 |  |  | 3 |  |  |  |  |  |
| **5008** | Case | 5 | 2 | 1 | 1 |  |  |  | 1 | F5L/F2 |
| **5009** | Case | 3 | 2 | 1 |  |  |  |  |  |  |
| **5010** | Case | 0 |  |  |  |  |  |  |  |  |
| **5011** | Ctrl | 7 | 1 | 1 | 3 | 1 | 1 | 1 | 1 | SMA |
| **5012** | Ctrl * | 5 | 2 | 1 | 2 |  |  |  |  |  |
| **5013** | Case | 3 | 2 | 1 |  |  |  |  |  |  |
| **5014** | Ctrl | 4 |  |  | 2 | 2 |  |  |  |  |
| **5015** | Case | 4 | 2 | 1 |  |  |  |  | 1 | F5L/F2, Methylenetetrahydrofolate reductase thermolabile variant 677C>T |
| **5016** | Case | 2 | 1 | 1 |  |  |  |  |  |  |
| **5017** | Case | 2 |  | 1 |  |  |  | 1 |  |  |
| **5018** | Ctrl | 1 |  |  | 1 |  |  |  |  |  |
| **5019** | Ctrl | 3 | 1 | 1 | 1 |  |  | 1 |  |  |
| **5020** | Ctrl | 8 | 2 | 1 | 4 |  |  | 3 | 1 | F5L/F2 |
| **5021** | Ctrl | 2 | 1 | 1 |  |  |  |  |  |  |
| **5022** | Ctrl | 4 | 1 | 1 | 2 |  |  | 1 |  |  |
| **5023** | Case | 2 |  |  | 2 |  |  | 2 |  |  |
| **5024** | Ctrl | 2 | 1 | 1 |  |  |  |  |  |  |
| **5025** | Ctrl | 3 | 1 | 1 | 1 |  |  |  |  |  |
| **5026** | Ctrl | 3 |  |  | 3 |  |  | 2 |  |  |
| **5027** | Ctrl | 2 | 1 | 1 |  |  |  |  |  |  |
| **5028** | Ctrl | 1 |  |  | 1 |  |  |  |  |  |
| **5029** | Ctrl* | 4 |  | 1 | 3 |  |  | 1 |  |  |
| **5030** | Case | 1 |  |  | 1 |  |  | 1 |  |  |
| **5031** | Ctrl | 2 | 1 | 1 |  |  |  |  |  |  |
| **5032** | Ctrl | 3 |  | 1 | 1 |  |  |  | 1 | F5L/F2 |
| **5033** | Case | 1 |  |  | 1 |  |  | 1 |  |  |
| **5034** | Case | 2 | 1 | 1 |  |  |  |  |  |  |
| **5035** | Case | 2 | 1 | 1 |  |  |  |  |  |  |
| **5036** | Case | 2 | 1 | 1 |  |  |  |  |  |  |
| **5037** | Ctrl | 4 | 3 | 1 |  |  |  |  |  |  |
| **5038** | Case | 2 | 1 | 1 |  |  |  |  |  |  |
| **5039** | Ctrl | 2 | 1 | 1 |  |  |  |  |  |  |
| **5040** | Ctrl* | 3 | 1 |  | 2 |  |  |  |  |  |
| **5041** | Ctrl | 4 | 1 | 1 | 1 |  |  |  | 1 | Sterol Quantification for Disorders of Cholesterol Synthesis |
| **5042** | Case | 7 | 1 | 1 | 1 | 1 | 1 |  | 3 | SMA; Plasma Total Lipids; Very Long Chain & Branched Fatty Acids; Oligosaccharide and glycan screening |
| **5043** | Case | 2 | 1 | 1 |  |  |  |  |  |  |
| **5044** | Ctrl | 1 |  |  | 1 |  |  |  |  |  |
| **5045** | Case | 5 | 3 | 1 | 1 |  |  |  |  |  |
| **5046** | Ctrl | 1 |  |  | 1 |  |  |  |  |  |
| **5047** | Case | 3 | 1 | 1 | 1 |  |  | 1 |  |  |
| **5048** | Case | 2 | 1 | 1 |  |  |  |  |  |  |
| **5049** | Case | 5 | 2 | 1 | 2 |  |  | 1 |  |  |
| **5050** | Case | 3 | 1 | 1 | 1 |  |  |  |  |  |
| **5051** | Case | 4 |  | 1 | 2 |  |  | 1 | 1 | F5L/F2 |
| **5052** | Ctrl | 1 |  | 1 |  |  |  |  |  |  |
| **5053** | Ctrl* | 6 | 1 | 1 | 2 | 1 |  | 1 | 1 | SMA |
| **5054** | Case | 2 |  | 1 | 1 |  |  |  |  |  |
| **5055** | Ctrl | 3 |  | 1 | 1 |  |  |  | 1 | 7-Dehydrocholesterol |
| **5056** | Ctrl | 2 |  |  | 2 |  |  | 1 |  |  |
| **5057** | Case | 3 | 1 | 1 | 1 |  |  |  |  |  |
| **5058** | Case | 2 |  | 1 | 1 |  |  | 1 |  |  |
| **5059** | Case | 5 | 2 | 1 | 1 |  |  |  | 1 | F5L/F2 |
| **5060** | Case | 0 |  |  |  |  |  |  |  |  |
| **5061** | Case | 6 | 1 | 1 | 2 | 1 | 1 |  | 1 | SMA |
| **5062** | Ctrl | 5 | 1 | 1 | 1 | 1 | 1 |  | 1 | SMA |
| **5063** | Ctrl | 3 | 1 | 1 |  |  |  |  | 1 | DEB |
| **5064** | Ctrl | 1 |  |  | 1 |  |  | 1 |  |  |
| Total Tests | | **201** | **53** | **48** | **65** | **8** | **5** | **23** | **27** |  |
| Mean Tests | | **3.1** | **26.4%** | **23.9%** | **32.3%** | **4.0%** | **2.5%** | **11.4%** | **13.4%** |  |
| Total Case Tests | | **89** | **30** | **26** | **22** | **3** | **3** | **10** | **8** |  |
| Mean Case Tests | | **2.8** | **33.7%** | **29.2%** | **24.7%** | **3.4%** | **3.4%** | **11.2%** | **9.0%** |  |
| Total Ctrl Tests | | **112** | **23** | **22** | **43** | **5** | **2** | **13** | **19** |  |
| Mean Ctrl Tests | | **3.4** | **20.5%** | **19.6%** | **38.4%** | **4.5%** | **1.8%** | **11.6%** | **17.0%** |  |

**Table S4: Comparison of measures of clinical utility of molecular diagnoses in cases (standard tests and rWGS) and controls (standard tests alone).** Actual clinical utility was measured by chart review of changes in management that occurred within 90 days of molecular diagnosis. It did not represent the maximum clinical utility based on literature review of potentially beneficial interventions associated with each diagnosis.

6

**Table S5: Times and methods of diagnosis and other factors related to primary and secondary outcomes.** DOL: day of life. Dx: Diagnosis. D/C: discharge. Std: Standard. WGS: rapid trio whole genome sequencing.
